# Endometriosis and Risk of Adverse Pregnancy Outcome: A Systematic Review and Meta-Analysis

**DOI:** 10.3390/jcm10040667

**Published:** 2021-02-09

**Authors:** Kjerstine Breintoft, Regitze Pinnerup, Tine Brink Henriksen, Dorte Rytter, Niels Uldbjerg, Axel Forman, Linn Håkonsen Arendt

**Affiliations:** 1 Department of Obstetrics and Gynecology, Aarhus University Hospital, 8200 Aarhus, Denmark; uldbjerg@clin.au.dk (N.U.); af@clin.au.dk (A.F.); lha@clin.au.dk (L.H.A.); 2 Department of Child and Adolescent Medicine, Aarhus University Hospital, 8200 Aarhus, Denmark; regitzepinnerup@gmail.com (R.P.); tine.brink.henriksen@clin.au.dk (T.B.H.); 3 Department of Public Health, Research Unit for Epidemiology, Aarhus University, 8000 Aarhus, Denmark; dr@ph.au.dk

**Keywords:** endometriosis, pre-eclampsia, preterm birth, cesarean section, small for gestational age, stillbirth, hemorrhage

## Abstract

Background: This systematic review and meta-analysis summarizes the evidence for the association between endometriosis and adverse pregnancy outcome, including gestational hypertension, pre-eclampsia, low birth weight, and small for gestational age, preterm birth, placenta previa, placental abruption, cesarean section, stillbirth, postpartum hemorrhage, spontaneous hemoperitoneum in pregnancy, and spontaneous bowel perforation in pregnancy. Methods: We performed the literature review in accordance with Preferred Reporting Items for Systematic Reviews and Meta-Analysis (PRISMA), by searches in PubMed and EMBASE, until 1 November 2020 (PROSPERO ID CRD42020213999). We included peer-reviewed observational cohort studies and case-control studies and scored them according to the Newcastle–Ottawa Scale, to assess the risk of bias and confounding. Results: 39 studies were included. Women with endometriosis had an increased risk of gestational hypertension, pre-eclampsia, preterm birth, placenta previa, placental abruption, cesarean section, and stillbirth, compared to women without endometriosis. These results remained unchanged in sub-analyses, including studies on spontaneous pregnancies only. Spontaneous hemoperitoneum in pregnancy and bowel perforation seemed to be associated with endometriosis; however, the studies were few and did not meet the inclusion criteria. Conclusions: The literature shows that endometriosis is associated with an increased risk of gestational hypertension, pre-eclampsia, preterm birth, placenta previa, placental abruption, cesarean section, and stillbirth.

## 1. Introduction

Endometriosis affects about 10% of women of reproductive age [1]. It is a chronic gynecological disease whereby estrogen-dependent endometrial fragments are located on organs other than the uterus, with the development of inflammation, adhesions, and pain [1], with the ovaries and the posterior cul-de-sac (pouch of Douglas) most often affected [2]. Several pathogenic mechanisms are possible, but the most supported is retrograde bleeding through the fallopian tubes, due to dysperistaltic uterine contractions [3]. Peritoneal levels of inflammatory mediators, such as cytokines, chemokines, and prostaglandins, increase, leading to a state of chronic inflammation in women with endometriosis [4].

Many pathogenic consequences of endometriosis have been suggested to play a role in the decreased fertility seen in these women, e.g., structural changes in the junctional zone, chronic inflammation, mechanical defects, and ovarian dysfunction [5,6]. Furthermore, abnormal remodeling of the spiral arteries in the junctional zone may cause defective deep placentation, thus increasing the risk of adverse pregnancy outcome [5].

Due to infertility, many women with endometriosis use assisted reproductive technology (ART) to conceive [7], which may increase the risk of adverse pregnancy outcome per se [8]. 

In recent years, many studies have investigated the association between endometriosis and pregnancy complications; however, the results have been ambiguous [9,10,11,12]. This systematic review and meta-analysis set out to evaluate the association between endometriosis and adverse pregnancy outcomes, including gestational hypertension, pre-eclampsia, low birth weight, small for gestational age (SGA), preterm birth, antepartum hemorrhage, placenta previa, placental abruption, cesarean section, stillbirth, and postpartum hemorrhage (PPH). We also reviewed studies on endometriosis related to spontaneous hemoperitoneum in pregnancy (SHiP) and spontaneous bowel perforation in pregnancy.

## 2. Materials and Methods

We performed the study in accordance with the Preferred Reporting Items for Systematic Reviews and Meta-Analysis (PRISMA) guidelines [13] (Supplementary Materials S1). The protocol was published to PROSPERO in November 2020. ID for protocol: CRD42020213999.

### 2.1. Information Sources 

We systematically searched PubMed and EMBASE from its inception until 1 November 2020, for all studies on endometriosis and adverse pregnancy outcome. The reference lists of the included studies were screened for additional literature. Prior to submission of the review (10 January 2021), an additional search was performed to ensure that no newer studies had been published since the latest literature search.

A priori, we decided to focus on the following outcomes: gestational hypertension, pre-eclampsia, low birth weight, SGA, preterm birth, placenta previa, placental abruption, cesarean section, stillbirth, PPH, SHiP, and spontaneous bowel perforation in pregnancy.

We used “MeSH” (PubMed) and “Emtree” (EMBASE) terms as well as free text words. The following MeSH-terms were used: endometriosis, adenomyosis, pre-eclampsia, pregnancy-induced hypertension, infant low birth weight, small for gestational age, premature birth, gestational age, placenta previa, abruptio placentae, hemoperitoneum, intestinal perforation, cesarean section, stillbirth, and postpartum hemorrhage. Please see the exact search strings in Supplementary Materials S2.

### 2.2. Study Selection and Data Extraction

Studies had to investigate the association between endometriosis and at least one of the predefined outcomes.

We included cohort studies and case-control studies. Thus, case reports, case series, commentaries, letters, editorials, and conference abstracts were excluded. No restrictions by language or time period were applied. However, studies in other languages than English were excluded during the full-text assessment.

Data extracted, by use of a structured extraction sheet, included information on bibliography and study design, characteristics of participants, information on confounders and intermediate factors, how endometriosis was diagnosed, how the reference group was recruited, definitions of outcomes, number and proportions per group with the specific outcome, and effect estimates, including crude or adjusted odds ratios (cOR or aOR), crude or adjusted relative risks (cRR or aRR), and 95% confidence intervals (CIs). Data extraction was performed by Kjerstine Breintoft (KB) and Regitze Pinnerup (RP) and in case of no consensus, a third author was consulted (Linn Håkonsen Arendt (LHA)). For cohort studies not providing risk estimates, we extracted information on numbers of outcomes among exposed and non-exposed.

### 2.3. Screening of Studies

Duplicates were removed, using EndNote (X9, Clarivate Analytics, Philadelphia, PA, U.S.) and Covidence (Veritas Health Innovation Ltd, Melbourne, Australia). Studies were screened by title and abstract by Axel Forman (AF) and KB. Any discrepancies were resolved by discussion and if in doubt a third author was consulted (LHA). Relevant studies were reviewed in full text by KB and RP, and any disagreements were resolved by discussion and by consulting a third author (LHA). 

### 2.4. Assessment of Bias 

The included studies were assessed by KB and RP, using the Newcastle–Ottawa quality assessment Scale (NOS). Consensus was reached by discussion in case of disagreement. In case of no consensus, a third author was consulted (LHA). NOS evaluates the studies based on the selection and comparability of the groups. Furthermore, the ascertainment of exposure and outcomes is assessed. A priori, based on directed Acyclic graphs (DAGs) and the existing literature, we selected the following relevant potential confounding factors: maternal age, smoking, body mass index (BMI), and socioeconomic status. These factors have been shown to be associated with endometriosis [14,15,16] and increase the risk of several adverse pregnancy outcomes [17,18,19,20]. Thus, we considered these the most important potential confounders. Adjustment for at least two of these resulted in one point, and adjustment for all resulted in two points. Studies could receive a score between zero and nine based on criteria defined a priori (Supplementary Materials S3). Adjustment, stratification, or sampling for ART did not result in a higher score. However, ART is discussed. 

### 2.5. Meta-Analyses

Most studies estimated the association by providing odds ratios or relative risks with 95% CIs. We used aORs or aRRs if available. EpiBasic (V4.4, Svend Juul and Morten Frydenberg, Aarhus, Denmark) was used to calculate odds ratios based on available data if odds ratios or relative risks were not provided. 

We used Review Manager [21] to conduct the meta-analyses. We used a random-effect inverse-variance weighted model providing a combined OR with 95% CI. Our main meta-analysis only included studies with a NOS score ≥7, as indicated in Table 1. To evaluate whether the results of the main analyses changed by including all studies regardless of study quality, a secondary analysis for each outcome was also conducted. Furthermore, to avoid the influence of ART on the associations, sub-analyses were performed including only studies investigating spontaneous conceived pregnancies in both the exposed and non-exposed groups.

Furthermore, we created funnel plots, using Software for Statistics and Data Science (STATA, 16, STATACorp LLC, Texas, TX, USA) to visualize the likelihood of publication bias if the number of studies was more than ten, in accordance with the Cochrane Handbook of Systematic Reviews [22]. The cohort studies without adverse outcomes in women with or without endometriosis were excluded from the funnel plots because odds ratios could not be calculated. 

### 2.6. Heterogeneity Assessment 

We assessed the heterogeneity between studies by considering the study characteristics including study design, setting, population, and definition of pregnancy outcome. Furthermore, in the meta-analyses, we used the I^2^ statistics to assess the statistical heterogeneity, as recommended by the Cochrane Handbook for Systematic Reviews threshold recommendations [23]. An I^2^ value of 0% to 40% suggests that the heterogeneity may not be important, 30% to 60% suggests moderate heterogeneity, 50% to 90% suggests substantial heterogeneity, and 75% to 100% suggests significant heterogeneity [23].

## 3. Results

**Figure 1:**
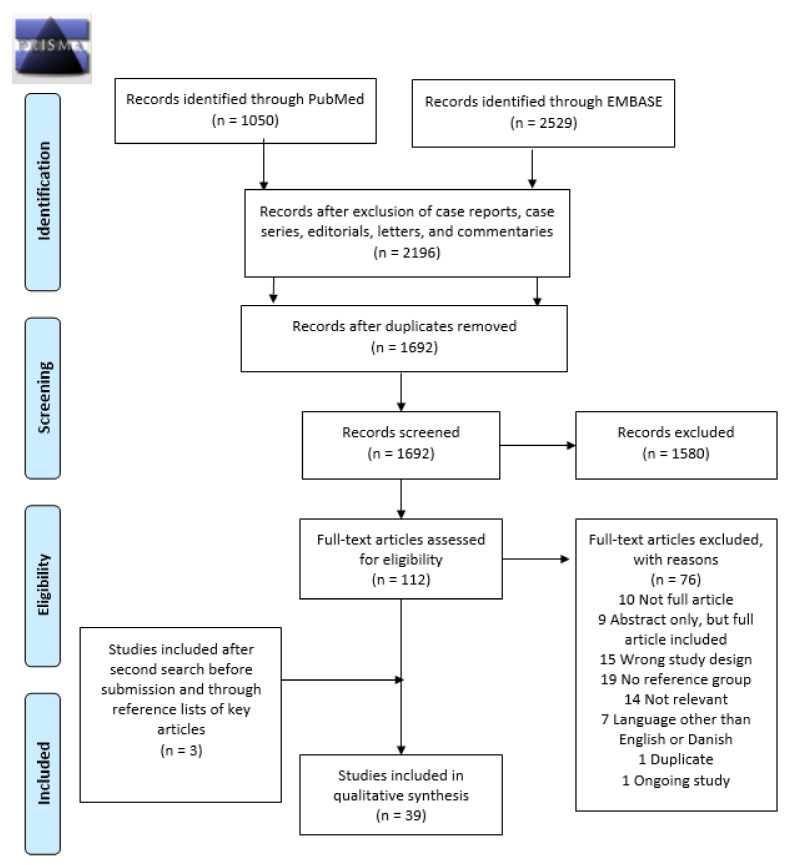
Preferred Reporting Items for Systematic Reviews and Meta-Analysis (PRISMA) flowchart identifying the inclusion of studies. Note: n, number.

A total of 1692 records were identified by the initial search. After screening titles and abstracts, 112 papers were reviewed in full text. This revealed 36 relevant papers. By scrutiny of their references, one additional paper was added. Prior to submission, an additional literature search revealed two relevant papers. A total of 39 cohort studies and no case-control studies were included in the systematic review. Figure 1 illustrates the PRISMA flowchart of study selection for the systematic review. Furthermore, characteristics and main results of the included studies are presented in Table 1 and Table 2.

For the outcomes SHiP and spontaneous bowel perforation in pregnancy, only one cohort study was available. The rest of the existing literature on these outcomes comprised of reviews, case reports, or case series. Thus, it was not possible to systematically review these outcomes. However, because of their severity, the existing literature was summarized and discussed. 

Fifteen studies received a NOS score ≥7 and were thus included in the main meta-analyses. The most frequent reasons for studies receiving a NOS score <7, and thus not being included in the main meta-analysis, were lack of adjustment for confounders, small sample size, comparison of pregnancies conceived by ART to spontaneous pregnancies, and inability to verify information on exposure and outcomes in medical records. The pooled ORs from the main meta-analyses were compared to the pooled ORs of the secondary analyses including all studies regardless of study quality. Six studies were eligible for the sub-analyses investigating only spontaneous pregnancies.

### 3.1. Heterogeneity Assessment

A priori, we considered the heterogeneity of the studies including sample size, country, timing, population, and data sources (Table 1). All studies were cohort studies.

Most studies originated from Italy, USA, Japan, or Scandinavia. Furthermore, the studies were conducted in varying time periods, from the 1970s, but with the majority published within the last five years (2015–2020). Data were mostly derived from medical records or registers. However, many of the studies also gathered information from questionnaires or did not state how information was collected. 

In the main meta-analyses, the I^2^ statistic was used to quantify the statistical heterogeneity and this revealed a high heterogeneity between studies for most outcomes, ranging from 0% to 96%. Gestational hypertension revealed an I^2^ of 0%; however, only four studies were included in the meta-analysis for this outcome [23].

### 3.2. Hypertensive Disorders of Pregnancy

Hypertensive disorders in pregnancy are associated with an increased risk of maternal and fetal morbidity and affect around 10% of all pregnant women worldwide [63]. A severe manifestation of this condition is pre-eclampsia which is characterized by hypertension, proteinuria, and maternal organ dysfunction after 20 weeks of gestation [64]. 

#### 3.2.1. Hypertensive Disorder in Pregnancy Overall

**Figure 2:**
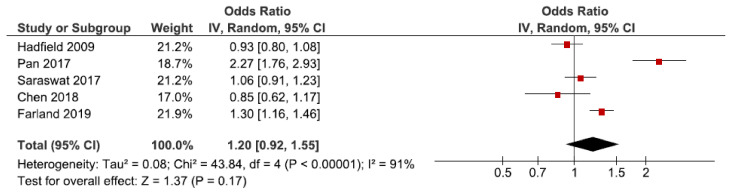
Forest plot for endometriosis and hypertensive disorders in pregnancy overall including studies with NOS ≥7. Chen et al., (2018) [30] and Farland et al., (2019) [34] used adjusted relative risks; Hadfield et al., (2009) [38], Pan et al., (2017) [51], and Saraswat et al., (2017) [53] used adjusted odds ratios.

A total of 11 cohort studies investigated hypertensive disorders in pregnancy overall [25,30,34,38,44,45,49,51,53,59,60]. Five were eligible for the main meta-analysis [30,34,38,51,53], without statistically significant difference between exposed and non-exposed to endometriosis (Figure 2). The main meta-analysis showed a pooled OR of 1.20 (95% CI: 0.92–1.55). Heterogeneity was high (I^2^ = 91%). The secondary analysis, including all studies regardless of study quality, did not change the direction of the estimated association between endometriosis and hypertensive disorders in pregnancy overall based on the high-quality studies (Supplementary Figure S1). Sub-analysis including only spontaneous pregnancies was not conducted as only one study was eligible [45].

The funnel plot was rather symmetrical (Supplementary Figure S2).

#### 3.2.2. Gestational Hypertension

**Figure 3:**
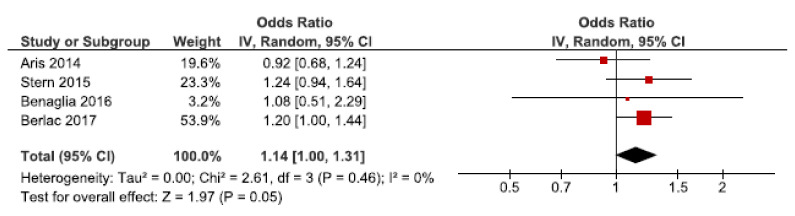
Forest plot for endometriosis and gestational hypertension, including studies with NOS ≥7. Aris et al., (2014) [24] and Benaglia et al., (2016) [27] used crude odds ratios; Berlac et al., (2017) [28] and Stern et al. (2015) [57] used adjusted odds ratios.

We identified 14 cohort studies on the association between endometriosis and gestational hypertension [24,27,28,29,31,33,46,47,48,50,52,55,57,61], four of which were included in the main-analysis [24,27,28,57] (Figure 3). Endometriosis showed to increase the risk of gestational hypertension with a pooled OR of 1.14 (95% CI: 1.00–1.31). Heterogeneity was low (I^2^ = 0%). The secondary analysis including all studies showed a pooled OR of 1.00 (95% CI: 0.79–1.27) (Supplementary Figure S3a). The sub-analyses including only spontaneous pregnancies showed similar results as the main meta-analysis, but with more uncertainty, as only three studies were included (Supplementary Figure S3b) [48,52,57]. 

The funnel plot was rather symmetrical (Supplementary Figure S4).

#### 3.2.3. Pre-Eclampsia

**Figure 4:**
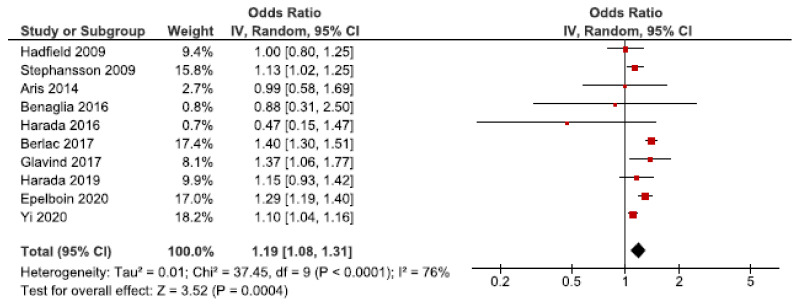
Forest plots for endometriosis and pre-eclampsia including studies with NOS ≥7. Aris et al., (2014) [24], Benaglia et al., (2016) [27], Hadfield et al., (2009) [38], and Harada et al., (2019) [40] used crude odds ratios; Berlac et al., (2017) [28], Epelboin et al., (2020) [32], Glavind et al., (2017) [37], Harada et al., (2016) [39], Stephansson et al., (2009) [56], and Yi et al., (2020) [62] used adjusted odds ratios.

We identified 21 cohort studies regarding endometriosis and pre-eclampsia [24,26,27,28,29,31,32,37,38,39,40,41,42,43,50,52,54,55,56,61,62]. Ten of these were included in the main meta-analysis [24,27,28,32,37,38,39,40,56,62] (Figure 4). Women with endometriosis showed to be at increased risk of pre-eclampsia (OR: 1.19, 95% CI: 1.08–1.31). Heterogeneity was high (I^2^ = 76%). Neither the secondary analysis including all studies regardless of quality nor the sub-analysis including only spontaneous pregnancies [32,52,54] changed the direction of the association between endometriosis and pre-eclampsia (Supplementary Figure S5).

The funnel plot was rather symmetrical (Supplementary Figure S6).

### 3.3. Low Birth Weight 

**Figure 5:**
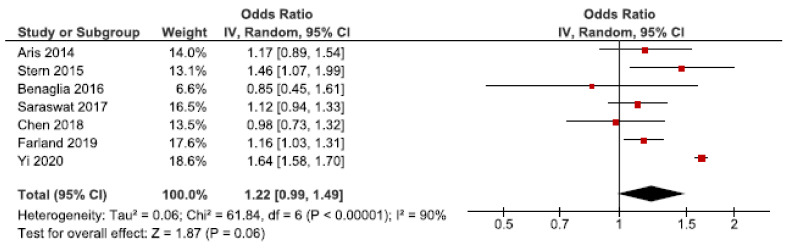
Forest plot for endometriosis and low birth weight, including studies with NOS ≥7. Chen et al., 2018 [30] and Farland et al., 2019 [34] used adjusted relative risks; Aris et al., (2014) [24] and Benaglia et al., (2016) [27] used crude odds ratios; Saraswat et al., (2017) [53], Stern et al., (2015) [57], and Yi et al., (2020) [62] used adjusted odds ratios.

Low birth weight may occur as a result of preterm birth or SGA [65]. We identified 14 cohort studies on endometriosis and low birth weight [24,26,27,30,34,42,43,46,49,52,53,57,58,62]. Seven were eligible for the main meta-analysis [24,27,30,34,53,57,62], without reaching statistically significance (Figure 5). We found a pooled OR of 1.22 (95% CI: 0.99–1.49). Heterogeneity was high I^2^ = 90%. Neither the secondary analysis including all studies nor the sub-analysis including only spontaneous pregnancies [52,57] changed the direction of the main results (Supplementary Figure S7).

The funnel plot did not indicate publication bias (Supplementary Figure S8).

### 3.4. Small for Gestational Age

**Figure 6:**
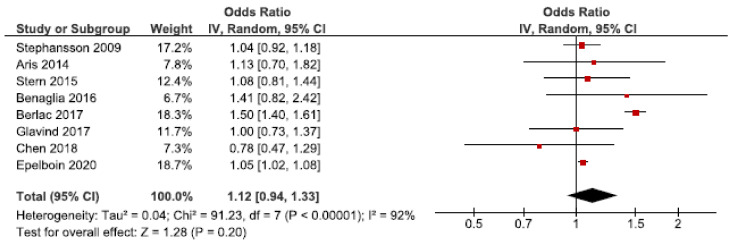
Forest plot for endometriosis and SGA, including studies with NOS ≥7. Chen et al., 2018 [30] used adjusted relative risk; Aris et al., 2014 [24] and Benaglia et al., 2016 [27] used crude odds ratios; Berlac et al., 2017 [28], Epelboin et al., 2020 [32], Glavind et al., 2017 [37], Stephansson et al., 2009 [56], and Stern et al., 2015 [57] used adjusted odds ratios.

Neonatal mortality and morbidity is increased in SGA infants [66]. We identified 22 cohort studies regarding the association between endometriosis and SGA [24,26,27,28,30,31,32,33,35,36,37,42,43,45,46,47,48,49,50,54,56,57]. Eight of these were included in the main meta-analysis [24,27,28,30,32,37,56,57], without reaching statistically significance (Figure 6). The pooled OR for the association between endometriosis and SGA was 1.12 (92% CI: 0.94–1.33). Heterogeneity was high (I^2^ = 92%). The secondary analysis, including all studies regardless of study quality, showed a pooled OR of 1.18 (1.02–1.36) (Supplementary Figure S9a). Five studies were eligible for the sub-analysis including only spontaneous pregnancies [32,45,48,54,57]. This showed an attenuated association with an OR of 1.05 (1.02–1.08) (Supplementary Figure S9b). Thus, the risk of SGA was only increased when including all studies regardless of study quality or only including spontaneous pregnancies. 

The funnel plot was rather symmetrical (Supplementary Figure S10).

### 3.5. Gestational Age at Birth and Preterm Birth

Gestational age is defined as the estimated time from the first day of the last menstrual period until birth, and preterm birth is defined as birth before 37 completed weeks of gestation [67]. The estimation was usually carried out by early pregnancy ultrasound scanning.

#### 3.5.1. Gestational Age at Birth

A total of 13 cohorts were found on endometriosis and gestational age at birth. Seven studies found women with endometriosis to have a shorter duration of pregnancy compared to women without endometriosis [31,39,40,41,43,55,60]. Two of these received a NOS score ≥7 [39,40]. Six studies found no difference in gestational age between women with and without endometriosis [25,42,44,49,54,61]. None of these received a NOS score ≥7. Ten of these studies also investigated preterm birth. No meta-analysis or funnel plot was performed for gestational age.

#### 3.5.2. Preterm Birth

**Figure 7:**
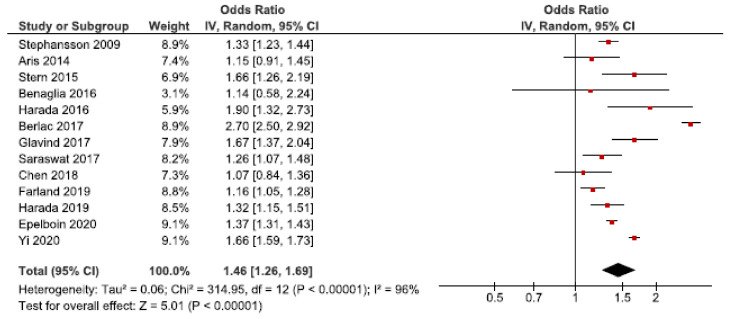
Forest plot for endometriosis and preterm birth including studies with NOS ≥7. Chen et al., 2018 [30] and Farland et al., 2019 [34] used adjusted relative risks; Aris et al., 2014 [24] and Harada et al., 2016 [39] used crude odds ratios; Benaglia et al., 2016 [27], Berlac et al., 2017 [28], Epelboin et al., 2020 [32], Glavind et al., 2017 [37], Harada et al., 2019 [40], Saraswat et al., 2017 [53], Stephansson et al., 2009 [56], Stern et al., 2015 [57], and Yi et al., 2020 [62] used adjusted odds ratios.

We identified 33 cohort studies on the association between endometriosis and preterm birth [24,25,26,27,28,30,31,32,33,34,35,36,37,39,40,41,42,43,44,45,46,47,48,49,50,52,53,56,57,58,59,61,62]. Thirteen of these were included in the main meta-analysis [24,27,28,30,32,34,37,39,40,53,56,57,62] (Figure 7). The pooled estimate showed an increased risk of preterm birth in women with endometriosis with an OR of 1.46 (95% CI: 1.26–1.69). Heterogeneity was high (I^2^ = 96%). Neither the secondary analysis including all studies regardless of quality nor the sub-analysis including only spontaneous pregnancies changed the direction of the results [32,45,48,52,57] (Supplementary Figure S11).

The funnel plot indicated no publication bias (Supplementary Figure S12). 

### 3.6. Antepartum Hemorrhage

Antepartum hemorrhage, including placenta previa and placental abruption, is one of the leading causes of maternal mortality worldwide [68].

The two large register-based studies by Stephansson et al. [56] and Berlac et al. [28] investigated all types of antepartum hemorrhage as one outcome. They both found endometriosis to be associated with antepartum hemorrhage.

#### 3.6.1. Placenta Previa

**Figure 8:**
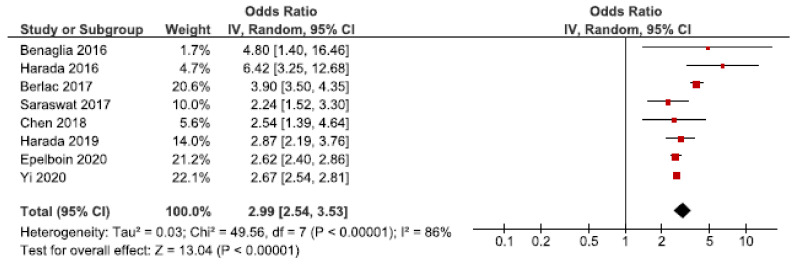
Forest plot for endometriosis and placenta previa including studies with NOS ≥7. Chen et al., 2018 [30] used adjusted relative risk; Benaglia et al., 2016 [27], Berlac et al., 2017 [28], Epelboin et al., 2020 [32], Harada et al., 2016 [39], Harada et al., 2019 [40], Saraswat et al., 2017 [53], and Yi et al., 2020 [62] used adjusted odds ratios.

Twenty-four cohort studies investigated the association between endometriosis and placenta previa [25,26,27,28,30,32,33,36,39,40,41,42,43,44,45,46,47,49,50,52,53,60,61,62]. Eight studies were eligible for the main meta-analysis [27,28,30,32,39,40,53,62] (Figure 8). It showed an increased risk of placenta previa in women with endometriosis with a pooled OR of 2.99 (95% CI: 2.54–3.53). Heterogeneity was high (I^2^ = 86%). Neither the secondary analysis including all studies regardless of quality nor the sub-analysis only including spontaneous pregnancies [32,45,52] changed the direction of the results (Supplementary Figure S13).

The funnel plot was rather symmetrical (Supplementary Figure S14).

#### 3.6.2. Placental Abruption

**Figure 9:**
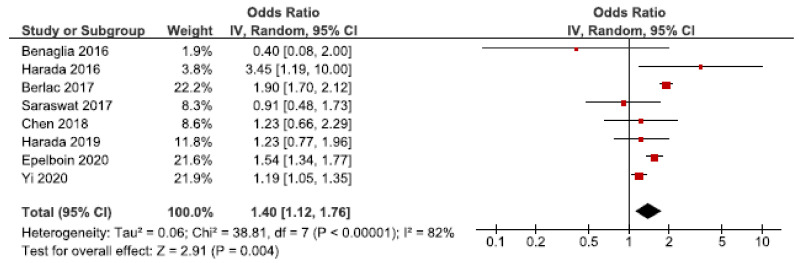
Forest plot for endometriosis and placental abruption including studies with NOS ≥7. Chen et al., 2018 [30] used adjusted relative risk; Benaglia et al., 2016 [27] and Harada et al., 2019 [40] used crude odds ratios; Berlac et al., 2017 [28], Epelboin et al., 2020 [32], Harada et al., 2016 [39], Saraswat et al., 2017 [53], and Yi et al., 2020 [62] used adjusted odds ratios.

Twenty cohort studies investigated placental abruption in endometriosis patients [26,27,28,30,32,33,39,40,42,43,44,45,46,49,50,52,53,59,61,62]. Eight studies were included in the main meta-analysis [27,28,30,32,39,40,53,62] (Figure 9). We found an increased risk of placental abruption in women with endometriosis with a pooled OR of 1.40 (95% CI: 1.12–1.76). Heterogeneity was high (I^2^ = 82%). Neither the secondary analysis including all studies or the sub-analysis only including spontaneous pregnancies changed the direction of the results (Supplementary Figure S15). However, in the sub-analysis the CIs were broad as two studies were included [32,45].

The funnel plot was rather symmetrical (Supplementary Figure S16).

### 3.7. Cesarean Section 

**Figure 10:**
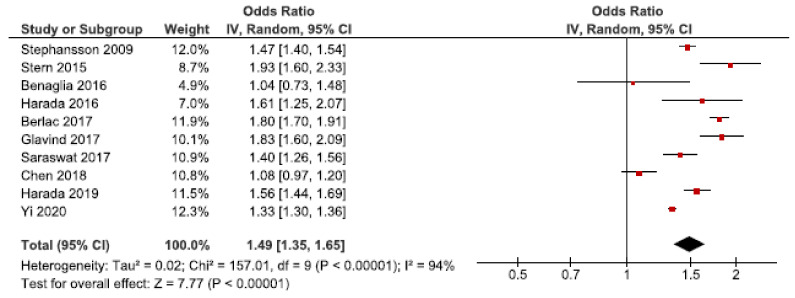
Forest plot for endometriosis and cesarean section including studies with NOS ≥7. Chen et al., 2018 [30] used adjusted relative risk; Benaglia et al., 2016 [27], Harada et al., 2016 [39], and Harada et al., 2019 [40] used crude odds ratios; Berlac et al., 2017 [28], Glavind et al., 2017 [37], Saraswat et al., 2017 [53], Stephansson et al., 2009 [56], Stern et al., 2015 [57], and Yi et al., 2020 [62] used adjusted odds ratios.

Cesarean section is indicated based on maternal (e.g., pre-eclampsia) or fetal complications (e.g., fetal distress). However, cesarean sections may cause severe maternal complications compared to vaginal deliveries [69]. We identified 28 cohort studies investigating the risk of cesarean section in women with endometriosis [25,26,27,28,30,31,33,37,39,40,41,42,44,45,46,47,48,49,50,52,53,55,56,57,59,60,61,62]. Ten of these were included in the main meta-analysis [27,28,30,37,39,40,53,56,57,62] (Figure 10), which showed an increased risk of cesarean section with an OR of 1.49 (95% CI: 1.35–1.65). Heterogeneity was high (I^2^ = 94%). Neither the secondary analysis including all studies regardless of study quality not the sub-analysis including only spontaneous pregnancies [45,48,52,57] changed the direction of the association between endometriosis and cesarean section (Supplementary Figure S17).

The funnel plot was rather symmetrical (Supplementary Figure S18).

### 3.8. Stillbirth 

**Figure 11:**
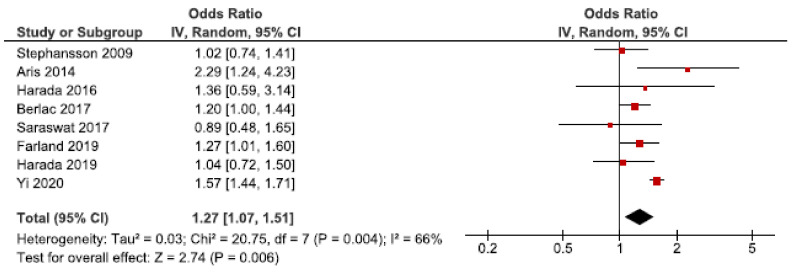
Forest plot for endometriosis and stillbirth including studies with NOS ≥7. Farland et al., 2019 [34] used adjusted relative risk; Aris et al., 2014 [24], Harada et al., 2016 [39], and Harada et al., 2019 [40] used crude odds ratios; Berlac et al., 2017 [28], Saraswat et al., 2017 [53], Stephansson et al., 2009 [56], and Yi et al., 2020 [62] used adjusted odds ratios.

In 2009, 2.64 million stillbirths were recorded across 42 countries [70]. We identified nine cohort studies regarding stillbirth [24,28,34,39,40,52,53,56,62]. Eight of these were eligible for the main meta-analysis [24,28,34,39,40,53,56,62], which showed an association between endometriosis and stillbirth (OR: 1.27, 95% CI: 1.07–1.51) (Figure 11). Heterogeneity was rather high (I^2^ = 66%). The secondary analysis including all studies did not change the direction of the results (Supplementary Figure S19). The sub-analysis, including only spontaneous pregnancies, was not conducted as only one study was eligible [52].

No funnel plot was made since only nine studies investigated stillbirth in women with endometriosis.

### 3.9. Postpartum Hemorrhage

**Figure 12:**
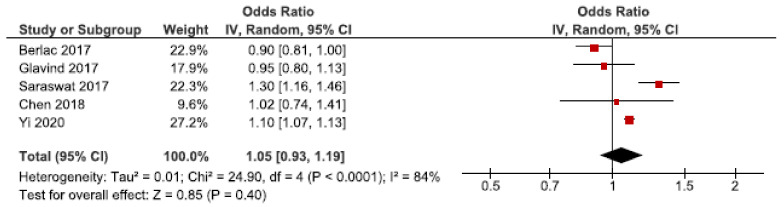
Forest plot for endometriosis and postpartum hemorrhage, including studies with NOS ≥7. Chen et al., 2018 [30] used adjusted relative risk; Berlac et al., 2017 [28], Glavind et al., 2017 [37], Saraswat et al., 2017 [53], and Yi et al., 2020 [62] used adjusted odds ratios.

Hemorrhage is the leading cause of maternal death worldwide, and PPH accounts for two-thirds of these [71]. We identified 15 cohort studies investigating the association between endometriosis and PPH [28,30,31,37,41,44,47,49,50,52,53,55,59,60,62]. Five of these were included in the main meta-analysis [28,30,37,53,62], without reaching statistically significance (Figure 12). We found a pooled OR of 1.05 (95% CI: 0.93–1.19). Heterogeneity was high (I^2^ = 84%). The secondary analysis, including all studies regardless of quality, did not change the direction of the association between endometriosis and PPH (Supplementary Figure S20). The sub-analysis, including only spontaneous pregnancies, was not conducted as only one study was eligible [52].

The funnel plot did not indicate publication bias (Supplementary Figure S21). 

### 3.10. Spontaneous Hemoperitoneum in Pregnancy

SHiP is a rare pregnancy complication associated with fetal and maternal mortality [10]. Exacoustos et al. conducted the only cohort study on SHiP in women with endometriosis [33]. They found the odds to be 24 times as high in women with endometriosis, compared to women without the disease (OR: 24.6, 95% CI: 1.15–528) [33], but this conclusion was based on two cases of SHiP in women with endometriosis and no cases in women without endometriosis [33]. Still, recent reviews support an increased but poorly defined risk of SHiP in women with endometriosis [10,72,73]. The review by Lier et al. found that 33 of 59 cases of SHiP occurred in women with endometriosis [73]. In the initial search for this review, another five case reports, including eight cases of SHiP associated with endometriosis, were identified [74,75,76,77,78]. Taken together, the available evidence indicates that endometriosis may be associated with increased risk of SHiP.

### 3.11. Spontaneous Bowel Perforation in Pregnancy

Spontaneous bowel perforation in pregnancy is an abdominal emergency [10]. Setúbal et al. reported on three cases of bowel perforation due to endometriosis, at their own center, as well as 12 cases through a search of the literature until 2013 [79]. A further three cases of spontaneous bowel perforation during pregnancy in endometriosis patients were reported in 2014 [80], 2016 [81], and 2018 [82]. All cases had the diagnosis proven either prior to pregnancy or through biopsies taken during surgery. Thus, available data indicate that spontaneous bowel perforation may occur with increased risk in patients with bowel endometriosis.

## 4. Discussion

In this systematic review and meta-analysis, we addressed the association between endometriosis and adverse pregnancy outcomes. Overall, existing evidence pointed towards an association between endometriosis and gestational hypertension, pre-eclampsia, preterm birth, placenta previa, placental abruption, cesarean section, and stillbirth. However, the results on low birth weight, SGA, and PPH showed no statistical significance, and future studies on these outcomes are encouraged. The literature on SHiP and bowel perforation in pregnancy was sparse but indicated that endometriosis seemed to increase the risk of these rare, but severe complications. 

### 4.1. Methodological Considerations 

When evaluating the results from this systematic review and meta-analyses, several methodological aspects of the included studies need to be addressed. Only cohort studies were included, and thus only methodological considerations regarding cohort studies were considered. We performed quality assessment of the studies, focusing on the risk of selection bias, information bias, and confounding. Studies with a NOS score ≥7 were included in the main meta-analysis and compared to the results from the secondary meta-analysis including all studies regardless of study quality and the results from the sub-analyses including only spontaneous pregnancies. Results from the meta-analyses including all studies regardless of study quality and the sub-analysis including only spontaneous pregnancies were similar to those of the meta-analyses only including studies with a NOS score ≥7. However, we consider the main meta-analysis with the high-quality studies most valid. 

A limitation of this systematic review is the high heterogeneity between the studies, which may explain, e.g., why hypertensive disorders in pregnancy, overall, had no statistically significant association with endometriosis, whereas gestational hypertension and pre-eclampsia did. This could well be due to the heterogeneous study populations of women with endometriosis, with various locations and types and severity of endometriosis. This review did not consider these aspects.

Selection bias is present if the association between endometriosis and adverse pregnancy outcomes differs between those included in the studies and the background population. Most studies did not report their participation rate, and among those that did, a rather small participation for women with endometriosis was reported [33,50]. A low participation rate may increase the risk of selection bias which could explain the different results found throughout the studies. Selection bias can be present if the frequency of adverse pregnancy outcome was higher in women with endometriosis who participated compared to women with endometriosis who did not participate in these studies, it could lead to bias away from the null.

Adjustment for potential confounders varied throughout the studies, and unadjusted or residual confounding may hamper interpretation of results. The covariates most often adjusted for were maternal age, parity, BMI, and smoking status. When scoring the original studies included in this review, we defined essential confounders as maternal age, socio-economic status, BMI, and smoking. We decided not to include parity in the NOS score because parity may be an intermediate factor between endometriosis and adverse pregnancy outcome. Adjusting for an intermediate factor may lead to bias towards the null and underestimate the association [83]. For pre-eclampsia, preterm birth, and stillbirth, the studies that adjusted for potential confounders tended to find a stronger association, as compared to the studies that did not adjust for any confounders. For placenta previa and cesarean section, most studies found an association regardless of adjustment for confounders. Furthermore, studies that adjusted for confounders tended to find a smaller association between endometriosis and adverse pregnancy outcome after adjustment

Women with endometriosis more often need fertility treatment, and ART may also affect pregnancy outcome. Thus, ART may act as an intermediate factor in the association between endometriosis and adverse pregnancy outcome [7,8].

Adjusting for ART may result in bias towards the null, thus leading to an underestimation of the association between endometriosis and adverse pregnancy outcomes [83]. The studies included handled ART in different ways, some by selection only within ART patients, while others adjusted, stratified, or excluded ART patients. Four studies included in this review adjusted for ART [28,30,31,49]. Berlac et al., showed a decreasing association when adjusting for ART between endometriosis and all outcomes apart from PPH [28]. The same pattern was seen in the studies by Chen et al., [30] and Miura et al., [49]. Conti et al., claimed that multivariate analysis including ART failed to change their results [31]. 

Two studies stratified their results by ART [37,56]. Glavind et al., found similar results in women with and without ART [37]. Stephansson et al., only stratified preterm birth by ART and found that the association attenuated in the ART group [56]. Furthermore, 14 of the included 37 studies neither stratified nor adjusted their results by ART.

The inclusion of women who conceived by ART or spontaneous pregnancies in only the exposed or non-exposed group may lead to bias. An association found when comparing ART pregnancies and spontaneous pregnancies might be due to ART and not endometriosis. Stern et al. [57] and Epelboin et al. [32] excluded ART pregnancies in their non-exposed group and divided their exposed group in ART and non-ART pregnancies. Exacoustos et al. only excluded ART pregnancies in their non-exposed group [33]. Lastly, Kuivasaari-Pirinen et al. [43] and Fernando et al. [35] compared ART pregnancies in endometriosis patients to spontaneous pregnancies in women without endometriosis. 

Epelboin et al. compared women with endometriosis spontaneously or by ART. They found ART to act as an independent risk factor for placenta previa, preterm birth and SGA [32]. However, data are limited, and more studies on this aspect are encouraged.

The classification of exposure varied across the studies and may be important to consider. Miura et al. included women diagnosed with endometriosis through symptoms [49] and Harada et al. from 2016 and 2019 gathered information on endometriosis from questionnaires [39,40]. This may have led to women without a verified diagnosis of endometriosis being included in the exposed group and thus non-differentiated misclassification and bias towards the null. The three studies all received a lower NOS score (Supplementary Materials S3).

Measurement error and misclassification of the outcomes may be present but is unlikely to depend on endometriosis status. The definitions of outcomes varied throughout the studies. Notably, regarding stillbirth, the studies which found an association all defined stillbirth as fetal loss occurring after 20 completed weeks of gestation, while in the studies that found no association included gestational ages ranged from 22 to 28 weeks and above. Furthermore, PPH was defined differently throughout the studies. However, these differences were present in both the studies that found an association and those that did not; thus, they should not alter the results. 

Additionally, not all studies stated how the outcome information was obtained [31,36,42,44,45,47,48]. Furthermore, three studies gathered information on outcomes by questionnaires and telephone interviews [25,27,34]. Self-reporting of outcomes may cause differential misclassification, as women with endometriosis might over-report adverse outcomes compared to women without endometriosis, which could then lead to an overestimation of the studied association. 

The external validity of several of the included studies may be limited as they were based on selected populations. Eight studies recruited their exposed group among women with previous endometriosis surgery [25,31,33,36,44,45,47,50]. Though this procedure ensures a verified diagnosis, these women no longer represent the general population of women with endometriosis. Another important aspect to consider is that several studies focused only on women who conceived spontaneously [45,48,52,54] or following ART [26,27,29,36,41,46,58]. We conducted sub-analyses only including spontaneous pregnancies, and, overall, these provided similar results. However, women with endometriosis who conceived spontaneously will most likely have milder forms of endometriosis compared to women with endometriosis who conceive by ART. If the severity of endometriosis affects the risk of adverse pregnancy outcome this might bias the results towards the null.

### 4.2. Potential Mechanisms

Various theories exist regarding both the increased risk of infertility and the increased risk of adverse pregnancy outcome in women with endometriosis. 

First, the thickening of the junctional zone, especially seen in women with advanced stages of endometriosis [84], may lead to abnormal remodeling of the spiral arteries and thus defective deep placentation [5,85]. The increased risk of placental dysfunction in women with endometriosis may lead to both adverse fetal outcomes (e.g., stillbirth) and adverse maternal outcomes (e.g., pre-eclampsia, placenta previa, placental abruption, and PPH) [5].

Furthermore, the inflammatory response caused by extrauterine endometrial cells may lead to increased levels of prostaglandins, cytokines, and macrophage activity, and by that to preterm labor contractions [86]. Additionally, the progesterone resistance and the increased estrogen levels seen in women with endometriosis may also contribute to an abnormal inflammatory response [87]. Furthermore, an increase in the activity of proteases and the breakdown of the extracellular matrix may lead to preterm pre-labor rupture of membranes (PPROM), and thus spontaneous preterm birth [86,88]. Although only sparsely studied, PPROM has previously been shown to be more frequent in women with endometriosis [31,39]. However, other studies failed to support this [27,30,39]. This could indicate that spontaneous preterm births, and not induced preterm births due to maternal or fetal complications, may be the main issue in endometriosis patients. This systematic review did not distinguish between spontaneous and induced preterm birth which may limit the results. Future studies on these aspects are encouraged. 

Adenomyosis is a condition, associated with endometriosis, in which the endometrium invades the myometrium. Adenomyosis has not been included in this review but could play a pathogenetic role since Kunz et al. found that 90% of women suffering from pelvic endometriosis also had a diagnosis of adenomyosis [89]. The thickened junctional zone is diagnostic for adenomyosis and can lead to a disrupted placental bed and thereby obstetrical complications [85,89]. A recent review showed adenomyosis to have an even stronger association with preterm birth and SGA than endometriosis [90].

The rare outcome SHiP is a potentially life-threatening condition during pregnancy. Lier et al. found that bleeding originated from endometriotic implants, ruptured utero-ovarian vessels, hemorrhagic nodules of decidualized tissue, or a combination of these [91] 

Spontaneous bowel perforations during pregnancy are mostly located in deep infiltrating bowel endometriosis in the sigmoid and rectum [92], and decidualization of these lesions represents a possible, yet unproven pathogenetic mechanism [79,93].

### 4.3. Clinical Aspects

The majority of original studies has rather consistently shown that women with endometriosis face a higher risk of preterm birth, irrespective of use of ART. However, future studies are needed to investigate preterm birth in more detail and to distinguish between medically indicated preterm birth and spontaneous preterm birth. Moreover, studies should investigate the effect of screening and potential interventions. 

Furthermore, the risk of placenta previa was substantially increased, and we encourage heightened awareness for this complication during pregnancy in women with endometriosis. 

The risk of cesarean section was also increased in women with endometriosis; however, this systematic review was limited by not differentiating between elective and acute cesarean section or investigating the underlying cause of cesarean section. Future studies are needed to determine whether the increased risk of cesarean sections is driven by acute or elective cesarean sections and whether they are carried out for fetal or maternal reasons in women with endometriosis.

The low incidence of SHiP and spontaneous bowel perforation in pregnancy implies that large-scale collaboration is needed to further define the risk profile and proper management of these serious pregnancy complications. With our present knowledge, an increased awareness in pregnant women with endometriosis seems reasonable when abdominal emergencies are encountered. 

## 5. Conclusions

This systematic review with meta-analyses supports the notion that endometriosis is associated with an increased risk of several adverse pregnancy outcomes, including gestational hypertension, pre-eclampsia, preterm birth, placenta previa, placental abruption, cesarean section, and stillbirth. Furthermore, the two severe complications, SHiP and spontaneous bowel perforation in pregnancy, may also be associated with endometriosis, but large observational studies are needed to explore this further.

## Figures and Tables

**Table 1:** Characteristics of the 39 included cohort studies investigating the association between endometriosis and adverse pregnancy outcome.

Author, Year	Country	Study Period	Sample Size	Study Population	Source of Exposure Data	Source of Outcome Data	ART	Exclusion Criteria	Type of Lesion	NOS Score
Aris et al., 2014 [24]	Canada	1997–2008	31,068 women 784 with endometriosis	Pregnancies in Canada	Medical records	Medical records	Both with and without ART No stratification or adjustment	Incomplete medical records, multiple births	All subtypes of endometriosis	7
Baggio et al., 2015 [25]	Italy	1996–2007	144 women 51 with endometriosis	Women who underwent surgery or delivered at hospital in Italy	Medical records	By contact	Both with and without ART No stratification or adjustment	Non-exposed: medical conditions, previous bowel surgery or suspicion of endometriosis, unable to contact	Colorectal segment resection du to endometriosis	4
Benaglia et al., 2012 [26]	Italy and Spain	2005–2009	234 women 78 with endometriosis	Pregnancies achieved by IVF or ICSI in Italy or Spain	Medical records	Medical records and questionnaires if data are missing	IVF or ICSI only	Biochemical pregnancies, ectopic pregnancies, multiple births	Ovarian endometriosis	6
Benaglia et al., 2016 [27]	Italy	2008–2014	478 women 239 with endometriosis	Pregnancies conceived by IVF or ICSI in Italy	Medical records	Questionnaires	IVF or ICSI only	Intrauterine malformation or fibroids, multiple births, pre-pregnancy diabetes or hypertension, organ transplant, antiphospholipid syndrome, chronic renal diseases, SLE, abnormal thyroid function	All subtypes of endometriosis	7
Berlac et al., 2017 [28]	Denmark	1977–2014	1,091,251 pregnancies 19,331 with endometriosis	Women giving birth in Denmark	The National Health Registry	The National Birth Registry	With and without ART Adjusted for ART	Age <15 years or >49 years	All subtypes of endometriosis	8
Brosens et al., 2007 [29]	Belgium	1991–2004	675 pregnancies 271 with endometriosis	Women attending an IVF-center in Ghent	Medical records	Medical records and questionnaires	IVF only	Exposed: other infertility diagnosis than endometriosis alone or male infertility Non-exposed: other causes for infertility than male causes	Pelvic endometriosis	4
Chen et al., 2018 [30]	Canada	2003–2013	52,202 women 469 with endometriosis	Pregnancies in Canada	Medical records	Medical records	With and without ART Adjusted for ART	History of fibroids, multiple births	All subtypes of endometriosis	8
Conti et al., 2015 [31]	Italy	Not stated	2,239 women 316 with endometriosis	Pregnancies attending gynecological and obstetrics units	Not stated Histologically verified	Not stated	With and without ART Adjusted for ART	Endocrine, autoimmune, systemic diseases and uterine disorders, multiple births, other races than Caucasian	Ovarian, peritoneal, and DIE	5
Epelboin et al., 2020 [32]	France	2013–2018	4,121,767 pregnancies 38,035 with endometriosis	Women giving birth in France	The French National Health System Database	The French National Health System Database	Non-exposed without ART Exposed divided in ART and spontaneous pregnancies	Birthweight <500 g, <22 weeks of gestation, frozen embryo transfer, multiple births Non-exposed: ART	All subtypes of endometriosis	7
Exacoustos et al., 2016 [33]	Italy	2011–2015	341 women 41 with endometriosis	Exposed: previous surgery Non-exposed: delivery during same time period	Medical records	Medical records and phone interviews, questionnaires	Non-exposed did not include ART No stratification or adjustment	Endocrine, autoimmune and systemic disease, uterine disorders	Remaining DIE nodule of 2 cm or more	5
Farland et al., 2019 [34]	USA	1989–2009	196,722 pregnancies 8875 with endometriosis	Registered nurses in the US	Questionnaires 96% verified in medical records	Questionnaires	With and without ART No stratification or adjustment	Year of pregnancy unknown, diabetes, cardiovascular disease or cancer prior to pregnancy, missing information on pregnancy	All subtypes of endometriosis	7
Fernando et al., 2009 [35]	Australia	1991–2004	1770 women 630 with endometriosis	Pregnancies in Australia	Medical records and register databases	Medical records and register databases	With and without ART No stratification or adjustment	Etiology of infertility, women conceiving at other fertility clinics, multiparous, multiple births	All subtypes of endometriosis	5
Fujii et al., 2016 [36]	Japan	2000–2014	604 women 92 with endometriosis	ART pregnancies at a facility in Japan	Medical records	Not stated	ART only	Suspected endometriosis, spontaneous pregnancy, endometrial or cervical cancer, conization, multiple births	All subtypes of endometriosis	6
Glavind et al., 2017 [37]	Denmark	1989–2013	82, 793 births 1719 with endometriosis	Women attending antenatal care at the Department of Obstetrics and Gynecology, Aarhus University Hospital	The Danish National Patient Registry and the Danish National Pathology Registry and Data Bank	The Aarhus Birth Cohort, the Danish Medical Birth Registry, and the Danish National Patient Registry	With and without ART Stratification for ART	Stillbirths, multiple births	All subtypes of endometriosis	8
Hadfield et al., 2009 [38]	Australia	2000–2005	208,879 women 3239 with endometriosis	Women with singleton births in Australia	Medical records	Midwives data collection, Department of Health Admitted Patient Data Collection	With and without ART No stratification or adjustment	Age <15 years or >45 years, miltiparous, multiple births	All subtypes of endometriosis	7
Harada et al., 2016 [39]	Japan	2011–2014	9186 women 330 with endometriosis	Pregnancies in Japan	Questionnaires	Medical records	With and without ARTNo stratification or adjustment	Incomplete covariate data, multiple births	All subtypes of endometriosis	7
Harada et al., 2019 [40]	Japan	2011–2014	96,655 pregnancies 3517 with endometriosis	Pregnancies in Japan	Questionnaires	Medical records	With and without ART No stratification or adjustment	Incomplete gynecological history, multiple births	All subtypes of endometriosis	7
Jacques et al., 2016 [41]	France	2009–2014	226 pregnancies 113 with endometriosis	Pregnancies within a reproductive care unit in France	Medical records and questionnaires if information was missing	Medical records and questionnaires if information was missing	ART only	Spontaneous miscarriage, ectopic pregnancy, <22 weeks of gestation, not able to be matched Exposed: other types of endometriosis than pelvic Non-exposed: female infertility	Pelvic endometriosis	5
Kortelahti et al., 2003 [42]	Finland	1994–2000	274 women 137 with endometriosis	Exposed: births at a hospital in Finland Non-exposed: tubal sterilization and laparoscopic surgery	Medical records	Not stated	With and without ART Matched for infertility treatment	Multiple births	All subtypes of endometriosis	6
Kuivasaari-Pirinen et al., 2012 [43]	Finland	1996–2007	26,919 pregnancies 49 with endometriosis	ART or ICSI pregnancies compared to spontaneous pregnancies in Finland	Birth registers	Birth registers	Endometriosis + ART compared to non-endometriosis + spontaneous	Birthweight <500 g, <22 weeks of gestation, pregnancies with major fetal malformations, multiparous, multiple births	All subtypes of endometriosis	6
Li et al., 2017 [44]	China	2011–2013	375 women 75 with endometriosis	Pregnancies at the Department of Obstetrics and Gynecology in Peking	Not stated Laparoscopic diagnosis	Not stated	With and Without ART No stratification or adjustment	Malignancies, adenomyosis, immune system, endocrine or cardiovascular disease, other complications, endometriosis not surgically verified, multiparous, multiple births	All subtypes of endometriosis	4
Lin et al., 2015 [45]	China	1995–2013	498 women 249 with endometriosis	Spontaneous pregnancies in China	Not stated Histologically verified	Not stated	Without ART	ART, malignancies, immune-system and cardiovascular disease, multiparous, multiple births	All subtypes of pelvic endometriosis	6
Luke et al., 2015 [46]	USA	2004–2008	2321 pregnancies 410 with endometriosis	ART pregnancies in Massachusetts Hospitals to Massachusetts women	SART CORS and PELL databases	SART CORS and PELL databases	ART only	Fetal death, >1 infertility diagnosis, triplet or quadruplet pregnancies, <20 weeks of gestation, birthweight <350g, spontaneous pregnancies	All subtypes of endometriosis	6
Mannini et al., 2017 [47]	Italy	2009–2014	786 women 262 with endometriosis	Women delivering at a tertiary hospital in Italy	Not stated Histologically verified	Not stated	With and without ART No stratification or adjustment	Biochemical or ectopic pregnancies, missing data	DIE, ovarian endometriosis, peritoneal endometriosis	5
Mekaru et al., 2014 [48]	Japan	1995–2011	88 women 40 with endometriosis	Pregnancies in Japan	Not stated Laparoscopic diagnosis	Not stated	Without IVF and embryo transfer No stratification or adjustment	IVF or embryo transfer pregnancies, hypertension or diabetes, age ≥41 years, multiple births	All subtypes of endometriosis	5
Miura et al., 2019 [49]	Japan	2010–2017	2769 women 80 with endometriosis	Pregnancies at Nagoya University Hospital in Japan	Medical records	Medical records	With and without ART Adjusted for ART	<22 weeks of gestation, fetal malformations, incomplete medical records, multiple births	All subtypes of endometriosis	5
Nirgianakis et al., 2018 [50]	Switzerland	2004–2016	248 women 62 with endometriosis	Pregnant women attending antenatal care at a University Hospital in Bern, Switzerland	Not stated Histologically verified	Medical records	With and without ART Matched for ART	Missing data, miscarriages in first trimester, ectopic pregnancies, multiple births Exposed: pregnancies before surgery, concomitant hysterectomy	DIE	4
Pan et al., 2017 [51]	Taiwan	1998–2012	12,890 women 2578 with endometriosis	Pregnancies in Taiwan	Longitudinal Health Insurance Database	Longitudinal Health Insurance Database	With and without ART No stratification or adjustment	Missing data, age <15 or >45 years, chronic hypertension, no record of prenatal visits 150 days before diagnosis of GH-PE, no blood pressure or urine protein to verify diagnosis of GH-PE Exposed: diagnosis of GH-PE prior to endometriosis diagnosis, no diagnosis of endometriosis in the reproductive age, inconsistent diagnosis of endometriosis	All subtypes of endometriosis	8
Porpora et al., 2020 [52]	Italy	2013–2019	425 women 145 with endometriosis	Spontaneous pregnancies in Italy	Not stated	Database not further clarified	Without ART	ART, intention of pregnancy termination, increased obstetrical risks, smoking, alcohol and/or drug addiction, other races than Caucasia, multiparous	Ovarian endometriosis, DIE, extra-pelvic endometriosis	6
Saraswat et al., 2017 [53]	Scotland	1981–2010	10,939 women 4232 with endometriosis	Pregnancies in Scotland	Medical records and health registers	Medical records	With and without ART No stratification or adjustment	Suspected diagnosis of endometriosis due to symptoms, multiple births	All subtypes of endometriosis	8
Scala et al., 2019 [54]	Italy	2017–2018	160 women 80 with endometriosis	Spontaneous pregnancies in Italy	Database not further clarified	Database not further clarified	Without ART	Adenomyosis, chronic hypertension, previous uterine surgery or malformations, autoimmune disease, fetal structural abnormalities and/or aneuploidy, ART, multiple births	DIE and ovarian endometriosis	6
Shmueli et al., 2019 [55]	Israel	2007–2014	61,535 births 135 with endometriosis	Pregnancies in a university-affiliated tertiary hospital in Israel	Medical records and database of the maternal-fetal unit and delivery room	Medical records and database of the maternal-fetal unit and delivery room	With and without ART No stratification or adjustment	Fetal and neonatal structural or chromosomal anomalies, multiple births	All subtypes of endometriosis	6
Stephansson et al., 2009 [56]	Sweden	1992–2006	1,442,675 births 13,090 with endometriosis	Pregnancies in Sweden	Swedish Medical Birth Register and patient register	Swedish Medical Birth Register	With and without ART Stratification for ART	Multiple births	All subtypes of endometriosis	9
Stern et al., 2015 [57]	USA	2004–2008	298,983 pregnancies 996 with endometriosis	Pregnancies in Massachusetts	SART CORS and PELL databases	SART CORS and PELL databases	Non-exposed without ART Exposed divided in ART and spontaneous pregnancies	Multiple infertility diagnoses, <20 weeks of gestation, birth weight <350g or >8165g, maternal age <18 years, not singleton or twin pregnancy Non-exposed: ART, previously subfertility	All subtypes of endometriosis	7
Sunkara et al., 2020 [58]	United Kingdom	1991–2016	40,794 pregnancies 5053 with endometriosis	Pregnancies conceived by IVF ± ICSI in the United Kingdom	The Human Fertilization and Embryology Authority	The Human Fertilization and Embryology Authority	IVF ± ICSI only	Missing data on gestational age or birth weight, spontaneous pregnancies, stillbirths, multiple births, donor insemination, embryo donation, preimplantation genetic diagnosis/screening, egg donation or freezing, gamete intra-Fallopian transfer + IVF or zygote intra-Fallopian transfer, embryos created for reasons other than infertility treatment, no fresh embryo transfer, unstimulated IVF, more than one cause of infertility		6
Tzur et al., 2018 [59]	Israel	1988–2013	502 women 35 with endometriosis	Pregnancies at a tertiary medical center in Israel	Medical records	Medical records	With and without ART No stratification or adjustment	Missing information, multiple births	All subtypes of endometriosis	6
Uccella et al., 2019 [60]	Italy	2011–2014	1808 women 118 with endometriosis	Pregnancies at the Department of Obstetrics and Gynecology of the University of Insubria	Medical records Database from the institution	Medical records Database from the institution	With and without ART No stratification or adjustment	Missing information on histological diagnosis and/or inaccurate description of endometriosis, multiparous	DIE, ovarian endometriosis, peritoneal endometriosis	6
Warzecha et al., 2020 [61]	Poland	2015–2018	360 women 64 with endometriosis	Pregnancies at University hospital in Warsaw, Poland	Medical records	Medical records	With and without ART No stratification or adjustment	<22 weeks of gestation, adenomyosis or other anatomical disorder, chronic diseases, multiple births	All subtypes of endometriosis	6
Yi et al., 2020 [62]	Korea	2007–2015	1,938,424 women 44,428 with endometriosis	Pregnancies in Korea	Korea National Health Insurance and the National Health Screening Program for Infants and Children	Korea National Health Insurance and the National Health Screening Program for Infants and Children	With and without ART No stratification or adjustment	Missing data on maternal age, no National Health Screening Program examination, multiparous	All subtypes of endometriosis	7

Abbreviations: ART, assisted reproductive technology; DIE, deep infiltrating endometriosis; GH-PE, gestational hypertensive preeclampsia; ICSI, intra cytoplasmatic sperm injection; IVF, in vitro fertilization; NOS, Newcastle–Ottawa Scale; PELL, Pregnancy to Early Life Longitudinal Data System; SART CORS, Society of Assisted Reproductive Technologies Clinical Outcomes Reporting System; SLE, systemic lupus erythematosus.

**Table 2:** Main results of the 39 included cohort studies investigating the association between endometriosis and adverse pregnancy outcome.

Author, Year	Outcome(s)	Crude or Adjusted OR (cOR or aOR) or Crude or Adjusted RR (cRR or aRR) with 95% CI	No OR Reported, But Reported Distribution(s) between Exposed and Non-Exposed *n* (%)	Number of Exposed vs. Non-Exposed	Confounder Adjustment
Aris et al., 2014 [24]	GH	cOR: 0.92 (0.68–1.24)		784 exposed	No adjustment for confounders
	PE	cOR: 0.99 (0.58–1.70)		31,068 non-exposed	
	LBW	cOR: 1.17 (0.89–1.54)			
	SGA	cOR: 1.13 (0.70–1.81)			
	PTB	cOR: 1.15 (0.91–1.45)			
	SB	cOR: 2.29 (1.24–5.22)			
Baggio et al., 2015 [25]	GH + PE	*cOR: 4.40 (1.24–15.66)	Exposed: 6 (20%), non-exposed: 5 (5.4%), *p* = 0.024	51 exposed	No adjustment for confounders
	GA (mean (SD))		Exposed: 38.1 weeks (3.3), non-exposed: 38.3 weeks (3.3), *p* = NS	93 non-exposed	
			Exposed: 6 (20%), non-exposed: 13 (14%), *p* = NS		
	PTB	*cOR: 1.54 (0.53–4.48)	Exposed: 2 (6.6%), non-exposed: 1 (1.1%), *p* = 0.045		
	PP	*cOR: 6.57 (0.57–75.21)	Exposed: 18 (60%), non-exposed: 27 (29%), *p* < 0.01		
	CS	*cOR: 3.67 (1.56–8.64)			
Benaglia et al., 2012 [26]	PE	*cOR: 2.05 (0.50–8.44)	Exposed 4 (5.1%), non-exposed 4 (2.6%)	78 exposed	Smoking, previous PTB, previous IVF-cycles, day 3 serum FSH
	LBW	aOR: 0.61 (0.20–1.86)		156 non-exposed	
	SGA	aOR: 0.56 (0.12–2.56)			
	PTB	aOR: 0.47 (0.14–1.54)			
	PP	*cOR: 3.08 (0.50–18.8)	Exposed: 3 (3.8%), non-exposed: 2 (1.3%)		
	PA		Exposed: 0, non-exposed: 3 (1.9%)		
	CS	aOR: 1.25 (0.63–2.50)			
Benaglia et al., 2016 [27]	GH	*cOR: 1.08 (0.51–2.29)	Exposed: 15 (7%), non-exposed: 14 (6%), *p* = 0.85	239 exposed	PTB and PP: BMI, duration of infertility
	PE	*cOR: 0.88 (0.31–2.45)	Exposed: 7 (3%), non-exposed: 8 (3%), *p* = 1.00	239 non-exposed	
	LBW	*cOR: 0.85 (0.45–1.58)	Exposed: 20 (9%), non-exposed: 24 (10%), *p* = 0.64		
	SGA	*cOR: 1.41 (0.82–2.43)	Exposed: 34 (15%), non-exposed: 26 (11%), *p* = 0.27		
	PTB	aOR: 1.14 (0.58–2.22)			
	PP	aOR: 4.80 (1.40–17.2)			
	PA	*cOR: 0.40 (0.08–2.06)	Exposed: 2 (1%), non-exposed: 5 (2%), *p* = 0.45		
	CS	*cOR: 1.04 (0.73–1.50)	Exposed: 108 (47%), non-exposed: 106 (45%), *p* = 0.64		
Berlac et al., 2017 [28]	GH	aOR: 1.2 (1.0–1.3)		19,331 exposed	Year of delivery, maternal age, parity, BMI, smoking, ART
	PE	aOR: 1.4 (1.3–1.5)		1,071,920 non-exposed	
	SGA	aOR: 1.5 (1.4–1.6)			
	PTB (<34 weeks)	aOR: 2.7 (2.5–2.9)			
	PTB (<28 weeks)	aOR: 3.1 (2.7–3.7)			
	APH	aOR 2.2 (2.0–2.5)			
	PP	aOR 3.9 (3.5–4.3)			
	PA	aOR: 1.9 (1.7–2.2)			
	CS (acute pre-labor)	aOR: 2.1 (2.0–2.3)			
	CS (planned)	aOR: 1.8 (1.7–1.8)			
	CS (acute in labor)	aOR: 1.8 (1.7–1.9)			
	SB	aOR: 1.2 (1.0–1.44)			
	PPH	aOR: 0.9 (0.9–1.0)			
Brosens et al., 2007 [29]	GH	*cOR: 0.38 (0.17–0.87)	Exposed: 8 (3.5%), non-exposed: 23 (8.7%)	271 exposed	Year and place of delivery
	PE	*cOR: 0.13 (0.03–0.58)	Exposed: 2 (0.8%), non-exposed: 16 (5.8%)	404 non-exposed	Matched for maternal age, parity, multiple pregnancies
Chen et al., 2018 [30]	GH + PE	aRR: 0.85 (0.62–1.15)		469 exposed	Maternal age, parity, neighborhood income, immigrant population, previous abortion, chronic hypertension, pre-existing diabetes, ART, infant sex
	LBW	aRR: 0.98 (0.73–1.31)		51,733 non-exposed	
	SGA	aRR: 0.78 (0.47–1.29)			
	PTB	aRR: 1.07 (0.84–1.37)			
	PP	aRR: 2.54 (1.39–4.64)			
	PA	aRR: 1.23 (0.66–2.29)			
	CS	aRR: 1.08 (0.97–1.20)			
	PPH	aRR: 1.02 (0.74–1.41)			
Conti et al., 2015 [31]	GH	*cOR: 0.62 (0.29–1.30)	Exposed: 8 (3.7%), non-exposed: 77 (5.8%)	316 exposed	SGA and PTB: infertility, ART
	PE	*cOR: 1.92 (0.70–5.30)	Exposed: 5 (2.2%), non-exposed 16 (1.2%)	1923 non-exposed	
	SGA	aOR: 2.72 (1.46–5.06)			
	GA (median)		Exposed: 39 weeks, non-exposed: 40 weeks, *p* = 0.0002		
	PTB	aOR: 2.24 (1.46–3.44)			
	CS	*cOR: 1.22 (0.89–1.67)	Exposed: 64 (29.1%), non-exposed: 337 (25.3%)		
	PPH	*cOR: 1.25 (0.76–2.05)	Exposed: 21 (9.4%), non-exposed: 104 (7.8%)		
Epelboin et al., 2020 [32]	PE (ART)	aOR: 1.11 (0.95–1.30)		38,035 exposed (6934 with ART and 31,101 without ART)	Maternal age, parity, smoking, diabetes, hypertensive disorders, obesity
	PE (no ART)	aOR: 1.29 (1.19–1.39)		4,083,732 non-exposed	SGA: gestational age and sex
	SGA (ART)	aOR: 1.25 (1.18–1.32)			
	SGA (no ART)	aOR: 1.05 (1.02–1.08)			
	PTB (ART)	aOR: 1.92 (1.78–2.07)			
	PTB (no ART)	aOR: 1.37 (1.31–1.43)			
	PP (ART)	aOR: 6.51 (5.82–7.28)			
	PP (no ART)	aOR: 2.62 (2.40–2.86)			
	PA (ART)	aOR: 1.87 (1.44–2.42)			
	PA (no ART)	aOR: 1.54 (1.34–1.77)			
Exacoustos et al., 2016 [33]	GH	cOR: 4.11 (1.45–11.7)		41 exposed	No adjustment for confounders
	SGA	cOR: 1.80 (0.58–5.64)		300 non-exposed	
	PTB (<37 weeks)	cOR: 6.87 (3.07–15.4)			
	PTB (<32 weeks)	cOR: 2.51 (0.49–12.9)			
	PP	cOR: 61.6 (7.35–516)			
	PA	cOR: 15.3 (1.36–173)			
	SHiP	cOR: 24.6 (1.15–528)			
	CS	cOR: 2.82 (1.40–5.65)			
Farland et al., 2019 [34]	GH + PE	aRR: 1.30 (1.16–1.45)		8875 exposed	Year of pregnancy, maternal age, parity, pregnancy interaction term, race, age at menarche, menstrual cycle length, BMI at age 18, smoking status, alcohol consumption, history of infertility
	LBW	aRR: 1.16 (1.03–1.29)		187,847 non-exposed	
	PTB	aRR: 1.16 (1.05–1.28)			
	SB	aRR: 1.27 (1.01–1.60)			
Fernando et al., 2009 [35]	SGA (OE + ART)	aOR: 1.95 (1.06–3.60)		630 exposed (95 with OE and 535 with other subtypes)	Year of delivery, parity, smoking
	SGA (others + ART)	aOR: 0.96 (0.68–1.38)		1140 non-exposed	Matched for year of delivery, maternal age
	PTB (OE + ART)	aOR: 1.98 (1.09–3.62)			
	PTB (others +ART)	aOR: 1.03 (0.70–1.53)			
Fujii et al., 2016 [36]	SGA	aOR: 1.43 (0.68–2.81)		92 exposed	Maternal age, parity, number of transferred embryos
	PTB	aOR: 2.08 (1.07–3.89)		512 non-exposed	
	PP	aOR: 15.1 (4.40–61.7)			
Glavind et al., 2017 [37]	PE	aOR: 1.37 (1.06–1.77)		1719 exposed	Year of delivery, maternal age, parity, BMI, maternal place of birth, years of school
	SGA	aOR: 1.00 (0.73–1.37)		81,074 non-exposed	
	PTB	aOR: 1.67 (1.37–2.05)			
	CS	aOR: 1.83 (1.60–2.09)			
	PPH	aOR: 0.95 (0.80–1.14)			
Hadfield et al., 2009 [38]	GH + PE	aOR: 0.93 (0.8–1.0)		3239 exposed	GH + PE: maternal age and weeks of gestation
	PE	cOR: 1.00 (0.8–1.2)		205,640 non-exposed	
Harada et al., 2016 [39]	PE (mild)	aOR: 0.47 (0.15–1.48)		330 exposed	PE, PP, and PA: maternal age, smoking, passive smoking, alcohol consumption
	PE (severe)	aOR: 1.25 (0.45–3.45)		8856 non-exposed	
	GA (median (range))		Exposed: 39 weeks (15–42.1), non-exposed: 39.3 weeks (7.4–42.3), *p* < 0.01		
			Exposed: 5 (1.5%), non-exposed: 78 (0.9%)		
	PTB (22–37 weeks)	*cOR: 1.90 (1.32–2.75)	Exposed: 34 (10.3%), non-exposed: 504 (5.7%)		
	PTB (<22 weeks)	*cOR: 2.50 (1.00–6.24)			
	PP	aOR: 6.42 (3.25–12.7)			
	PA	aOR: 3.45 (1.19–10.0)	Exposed: 85 (25.8%), non-exposed: 1570 (17.7%), *p* < 0.01 Exposed: 6 (1.8%), non-exposed: 119 (1.3), *p* = 0.46		
	CS	*cOR: 1.61 (1.25–2.07)			
			
	SB/abortion	*cOR: 1.36 (0.59–3.11)			
Harada et al., 2019 [40]	PE (mild)	*cOR: 1.15 (0.93–1.42)	Exposed: 91 (2.6%), non-exposed: 2099 (2.3%), *p* = 0.204	3517 exposed	PTB and PP: maternal age, smoking, passive smoking, alcohol consumption
			Exposed: 42 (1.2%), non-exposed: 881 (1.0%), *p* = 0.133	93,138 non-exposed	
	PE (severe)	*cOR: 1.27 (0.93–1.73)	Exposed: 39 weeks (10–42), non-exposed: 39 weeks (6–43), *p* < 0.001		
	GA (median (range))				
			
			
	PTB (28–36 weeks)	aOR: 1.32 (1.15–1.53)	Exposed: 19 (0.5%), non-exposed: 410 (0.4%), *p* = 0.364		
	PTB (22–27 weeks)	aOR: 1.97 (1.26–3.09)	Exposed: 915 (26.1%), non-exposed: 17,151 (18.5%), *p* < 0.001		
	PP	aOR: 2.87 (2.19–3.75)	Exposed: 31 (0.9%), 791 (0.9%), *p* = 0.779		
	PA	*cOR: 1.23 (0.77–1.95)			
	CS	*cOR: 1.56 (1.44–1.68)			
			
	SB/abortion	*cOR: 1.04 (0.72–1.49)			
Jacques et al., 2016 [41]	PE	cOR: 8.53 (1.05–69.40)		113 exposed	Matched for maternal age, singleton or twin pregnancy, primary or secondary infertility, IVF with or without ICSI
	GA (mean (SD))		Exposed: 38.6 weeks (3.11), non-exposed: 39.4 weeks (2.26), *p* = 0.04	113 non-exposed	
	PTB	cOR: 2.05 (1.01–4.16)			
			
	PP	cOR: 1.0 (0.20–5.06)cOR: 2.64 (1.37–5.07)			
	CS	cOR: 1.0 (0.40–2.50)			
	PPH				
Kortelahti et al., 2003 [42]	PE	*cOR: 0.57 (0.24–1.36)	Exposed: 9 (6.6%), non-exposed: 15 (11.0%), *p* = 0.20	137 exposed	Matched for IVF-status and parity
	LBW	aOR: 1.01 (0.41–2.45)		137 non-exposed	LBW, SGA, and PTB: maternal age
	SGA	aOR: 1.09 (0.46–2.57)			
	GA (mean (SD))		Exposed: 276 days (14), non-exposed: 274 days (20), *p* = 0.531		
			
	PTB	aOR: 0.84 (0.38–1.88)	Exposed: 6 (4.4%), non-exposed: 4 (2.9%), *p* = 0.749		
	PP	*cOR: 1.52 (0.42–5.52)	Exposed: 3 (2.2%), non-exposed: 1 (0.7%), *p* = 0.622		
	PA	*cOR: 3.04 (0.31–29.64)	Exposed: 43 (31.4%), non-exposed: 42 (30.7%), *p* = 0.896		
	CS	*cOR: 1.03 (0.62–1.73)			
Kuivasaari-Pirinen et al., 2012 [43]	PE	*cOR: 1.14 (0.28–4.70)	Exposed: 2 (4.1%), non-exposed: 967 (3.6%), *p* = NS	49 exposed	LBW, SGA, and PTB: Age, parity, BMI, smoking, previous fetal deaths, (previous) miscarriages, chronic illness, marital status
	LBW	aOR: 2.13 (0.84–5.41)		26,870 non-exposed	
	SGA	aOR: 0.49 (0.15–1.59)			
	GA (mean (SD))		Exposed: 268 days (23), non-exposed: 277 days (15), *p* < 0.05		
			
	PTB	aOR: 3.25 (1.50–7.07)	Exposed: 3 (6.1%), non-exposed: 161 (0.6%), *p* < 0.005		
	PP	*cOR: 10.9 (3.34–35.3)	Exposed: 0, non-exposed: 161 (0.6%), *p* = NS		
	PA				
Li et al., 2017 [44]	GH + PE	aOR: 0.47 (0.10–2.34)		75 exposed	Maternal age at delivery, parity
	GA (median (IQR))		Exposed: 39 weeks (38–40), non-exposed: 39 weeks (38–40), *p* = 0.188	300 non-exposed	
			
	PTB	aOR: 1.30 (0.34–4.25)			
	PP	aOR: 0.56 (0.08–4.10)			
	PA	aOR: 1.39 (0.68–2.85)			
	CS	aOR: 1.53 (0.83–2.84)			
	PPH	aOR: 2.27 (1.06–4.87)			
Lin et al., 2015 [45]	GH + PE	aOR: 0.78 (0.31–2.00)		249 exposed	Maternal age
	SGA	aOR: 1.75 (0.41–7.49)		249 non-exposed	
	PTB	aOR: 2.42 (1.05–5.57)			
	PP	aOR: 4.51 (1.23–16.5)			
	PA	aOR: 0.98 (0.71–1.34)			
	CS	aOR: 1.93 (1.31–2.84)			
Luke et al., 2015 [46]	GH	aOR: 0.61 (0.41–0.89)		410 exposed (295 singletons)	GH, LBW, SGA, PTB, and CS: maternal and paternal demographic factors, plurality at birth, maternal preexisting medical conditions, ART factors
	LBW	aOR: 0.71 (0.50–1.01)		1911 non-exposed (1411 singletons)	
	SGA	aOR: 0.69 (0.44–1.07)			
	GA (mean (SD))		Exposed: 38.5 weeks (2.1), non-exposed: 38.6 weeks (1.9)		
			
	PTB	aOR: 1.02 (0.75–1.39)	Exposed: 10 (2.4%), non-exposed: 31 (1.6%)		
	PP (singleton)	*cOR: 1.52 (0.74–3.12)	Exposed: 9 (2.1%), non-exposed: 36 (1.9%)		
	PA (singleton)	*cOR: 1.17 (0.56–2.45)			
	CS	aOR: 1.11 (0.84–1.46)			
Mannini et al., 2017 [47]	GH	*cOR: 1.30 (0.58–2.91)	Exposed: 11 (4.2%), non-exposed: 14 (2.7%)	262 exposed	No adjustment for confounders
	SGA	*cOR: 1.56 (0.84–2.89)	Exposed: 19 (7.3%), non-exposed: 25 (4.8%)	524 non-exposed	
	PTB	*cOR: 3.10 (1.92–5.03)	Exposed: 44 (16.8%), non-exposed: 32 (6.1%)		
	PP	*cOR: 3.43 (1.23–9.53)	Exposed: 10 (3.8%), non-exposed: 6 (1.1%)		
	CS	*cOR: 2.32 (1.71–3.14)	Exposed: 149 (56.9%), non-exposed: 190 (36.3%)		
	PPH	*cOR: 0.89 (0.52–1.50)	Exposed: 22 (8.4%), non-exposed: 49 (9.4%)		
Mekaru et al., 2014 [48]	GH	*cOR: 1.24 (0.37–4.18)	Exposed: 6 (15%), non-exposed: 6 (12.5%), *p* = 0.73	40 exposed	No adjustment for confounders
	SGA	*cOR: 1.21 (0.07–18.58)	Exposed: 1 (2.5%), non-exposed: 1 (2.1%), *p*= 0.56	48 non-exposed	
	GA (mean (SD))		Exposed: 38.9 weeks (1.5), non-exposed 38.8 weeks (1.7), *p* = 0.72		
			Exposed: 3 (7.5%), non-exposed: 4 (8.3%), *p* = 0.8		
	PTB	*cOR: 0.89 (0.19–4.24)	Exposed: 13 (32.5%), non-exposed: 11 (22.9%), *p* = 0.31		
	CS	*cOR: 1.62 (0.63–4.16)			
Miura et al., 2019 [49]	GH + PE	*cOR: 0.70 (0.25–1.95)	Exposed: 4 (5.0%), non-exposed: 187 (7.0%), *p* = 0.66	80 exposed	PP: maternal age, parity, BMI, ART
	LBW	*cOR: 1.29 (0.70–2.36)	Exposed: 13 (16.2%), non-exposed: 352 (13.1%), *p* = 0.51	2689 non-exposed	PPH: maternal age, parity, placenta previa, macrosomia, BMI, ART
			Exposed: 2 (2.5%), non-exposed: 98 (3.6%), *p* = 1.00		
	SGA	*cOR: 0.68 (0.16–2.80)	Exposed: 38.3 weeks (2.1), non-exposed: 38.4 weeks (2.4), *p* = 0.34		
	GA (mean (SD))		Exposed: 8 (10%), non-exposed: 322 (12%), *p* = 0.72		
			
			Exposed: 2 (2.5%), non-exposed: 17 (0.6%), *p* = 0.10		
	PTB	*cOR: 0.82 (0.39–1.71)	Exposed: 30 (37.5%), non-exposed 681 (25.3%)		
	PP	aOR: 3.19 (1.56–6.50)	Exposed: 13 (16.2%), non-exposed 496 (18.4%)		
	PA	*cOR: 3.88 (0.88–17.08)			
	CS (scheduled)	*cOR: 1.77 (1.12–2.81)			
	CS (emergency)	*cOR: 0.86 (0.47–1.57)			
	PPH	aOR: 1.14 (0.66–1.98)			
Nirgianakis et al. 2018 [50]	GH	cRR: 6.00 (1.13–32.0)		62 exposed	Matched for maternal age, parity, mode of conception, CS history
	PE	cRR: 1.80 (0.44–7.32)		186 non-exposed	
	SGA	cRR: 1.62 (0.68–3.87)			
	PTB	cRR: 1.82 (0.79–4.20)			
	PP		Exposed: 4 (6.5%), non-exposed: 0, *p* = 0.004		
	PA		Exposed: 1 (1.6%), non-exposed: 0, *p* = NS		
	CS (primary)	cRR: 1.54 (0.98–2.43)			
	PPH	cRR: 1.88 (0.90–3.92)			
Pan et al., 2017 [51]	GH + PE	aOR: 2.27 (1.76–2.93)		2578 exposed	Maternal age. Age at diagnosis, occupation, urbanization, economic status, comorbidities
				10,312 non-exposed	
Porpora et al., 2020 [52]	GH	*cOR: 0.84 (0.34–2.08)	Exposed: 7 (5%), non-exposed: 16 (6%), *p* = NS	145 exposed	No adjustment for confounders
	PE	*cOR: 2.94 (0.49–17.8)	Exposed: 3 (2%), non-exposed: 2 (1%), *p* = NS	280 non-exposed	
	LBW (1500 g–2500 g)	*cOR: 0.74 (0.33–1.64)	Exposed: 9 (8%), non-exposed: 23 (9%), *p* = NS		
	LBW (<1500 g)	*cOR: 2.56 (0.65–9.32)	Exposed: 5 (4%), non-exposed: 4 (2%), *p* = NS		
	PTB	*cOR: 3.86 (2.08–7.14)	Exposed: 29 (20%), non-exposed: 21 (8%), *p* = 0.001		
	PP	*cOR: 2.67 (0.59–12.1)	Exposed: 4 (3%), non-exposed: 3 (1%), *p* = NS		
	PA		Exposed: 2 (1%), non-exposed: 0, *p* = NS		
	CS	*cOR: 1.59 (1.01–2.51)	Exposed: 51 (35%), non-exposed: 87 (31%), *p* = 0.042		
	SB	*cOR: 3.90 (0.35–43.4)	Exposed: 2 (1%), non-exposed: 1 (0.4%), *p* = NS		
	PPH	*cOR: 2.62 (0.58–11.9)	Exposed: 4 (3%), non-exposed: 3 (1%), *p* = NS		
Saraswat et al., 2017 [53]	GH + PE	aOR: 1.06 (0.91–1.24)		4232 exposed	Year of pregnancy, maternal age, parity, SES
	LBW	aOR: 1.12 (0.94–1.32)		6707 non-exposed	
	PTB	aOR: 1.26 (1.07–1.49)			
	PP	aOR: 2.24 (1.52–3.31)			
	PA	aOR: 0.91 (0.48–1.74)			
	CS	aOR: 1.40 (1.26–1.55)			
	SB	aOR: 0.89 (0.48–1.66)			
	PPH	aOR: 1.30 (1.16–1.46)			
Scala et al., 2019 [54]	PE (OE)	*cOR: 1.37 (0.36–5.16)	Exposed: 4 (10%), non-exposed: 6 (7.5%), *p* = 0.640	80 exposed (40 with OE and 40 with DE)	SGA: maternal age, ethnicity, BMI, PAPP-A
	PE (DE)	*cOR: 1.00 (0.24–4.23)	Exposed: 9 (7.5%), non-exposed: 6 (7.5%), *p* = 1.00	80 non-exposed	
	SGA (OE)	aOR: 1.49 (0.37–6.07)			
	SGA (DE)	aOR: 2.12 (0.43–10.6)			
	GA (OE)		Exposed: 39.1 weeks (38.0–40.5), non-exposed: 39.0 weeks (38.1–40.5), *p* = 0.93		
	(median (IQR))		Exposed: 39.2 weeks (38.1–40.5), non-exposed: 39.0 weeks (38.1–40.5), *p* = 0.81		
			
	GA (DE)				
	(median (IQR))				
Shmueli et al., 2019 [55]	GH	*cOR: 1.04 (0.26–4.20)	Exposed: 2 (1.5%), non-exposed: 877 (1.4%), *p* = 0.96	135 exposed	CS and PPH: maternal age and parity
	PE (mild)	*cOR: 0.86 (0.12–6.16)	Exposed: 1 (0.7%), non-exposed: 528 (0.9%), *p* = 0.88	61,400 non-exposed	
	PE (severe)	*cOR: 3.01 (0.74–12.2)	Exposed: 2 (1.5%), non-exposed: 305 (0.5%), *p* = 0.10		
	GA (mean (SD))		Exposed: 37.8 weeks (2.0), non-exposed: 38.9 weeks (1.8), *p* < 0.001		
			
	CS	aOR: 5.01 (3.34–7.52)			
	PPH	aOR: 3.70 (1.60–8.53)			
Stephansson et al., 2009 [56]	PE	aOR: 1.13 (1.02–1.26)		13,090 exposed	Year of delivery, maternal age, BMI, smoking, parity, years of formal education
	SGA	aOR: 1.04 (0.92–1.17)		1,429,585 unexposed	
	PTB	aOR: 1.33 (1.23–1.44)			
	APH	aOR: 1.76 (1.56–1.99)			
	CS	aOR: 1.47 (1.40–1.54)			
	SB	aOR: 1.02 (0.74–1.40)			
Stern et al., 2015 [57]	GH (ART)	aOR: 0.90 (0.64–1.26)		996 exposed (406 with ART and 590 without ART)	Maternal age, plurality, race and ethnicity, education, chronic hypertension, pre-pregnancy diabetes mellitus
	GH (no ART)	aOR: 1.24 (0.94–1.63)		297,987 non-exposed	
	LBW (ART)	aOR: 0.97 (0.70–1.33)			
	LBW (no ART)	aOR: 1.46 (1.07–1.99)			
	SGA (ART)	aOR: 1.05 (0.77–1.43)			
	SGA (no ART)	aOR: 1.08 (0.81–1.43)			
	PTB (ART)	aOR: 1.22 (0.90–1.66)			
	PTB (no ART)	aOR: 1.66 (1.26–2.18)			
	CS (ART)	aOR: 2.12 (1.67–2.69)			
	CS (no ART)	aOR: 1.93 (1.60–2.33)			
Sunkara et al., 2020 [58]	LBW	aOR: 1.11 (0.96–1.30)**		5053 exposed	Maternal age, year of treatment, previous live birth, IVF or ICSI, number of embryos transferred, fresh or frozen cycle
	PTB	aOR: 1.17 (1.01–1.35)**		35,741 non-exposed	
Tzur et al., 2018 [59]	GH + PE	*cOR: 0.82 (0.19–3.59)	Exposed: 2 (5.7%), non-exposed: 32 (6.9%), *p* = 0.80	35 exposed	PTB: maternal age, previous CS, hypertension disorders, PROM, GDM, PP, IVF
	PTB	aOR: 0.79 (0.27–2.35)	Exposed: 8 (22.9%), non-exposed: 41 (8.8%), *p* = 0.01	467 non-exposed	CS: maternal age, previous CS, IUGR, GDM, hypertension disorders
	PA	*cOR: 1.69 (0.21–13.9)	Exposed: 1 (2.9%), non-exposed: 8 (1,7%), *p* = 0.62		
	CS	aOR: 38.1 (11.0–131)			
	PPH		Exposed: 0, non-exposed: 3 (0.6%), *p* = 1.00		
Uccella et al., 2019 [60]	GH + PE	*cOR: 1.99 (1.08–3.67)	Exposed: 13 (11%), non-exposed: 99 (5.9%), *p* = 0.04	118 exposed	No adjustment for confounders
	GA (median (range))		Exposed: 38.9 weeks (29.9–42), non-exposed: 39.6 weeks (23.3–42.1), *p* < 0.001	1690 non-exposed	
			Exposed: 4 (3.4%), non-exposed: 8 (0.5%), *p* = 0.006		
	PP	*cOR: 7.38 (2.19–24.87)	Exposed: 49 (41.5%), non-exposed: 409 (24.2%), *p* < 0.0001		
	CS	*cOR: 2.22 (1.52–3.26)	Exposed: 21 (17.8%), non-exposed: 413 (24.4%), *p* = 0.051		
			
	PPH	*cOR: 0.57 (0.41–1.09)			
Warzecha et al., 2020 [61]	GH	cOR: 0.8 (0.3–2.2)		64 exposed	No adjustment for confounders
	PE	cOR: 0.7 (0.1–5.4)		296 non-exposed	
	GA (mean (SD))		Exposed: 38.6 weeks (1.6), non-exposed: 38.7 weeks (2.0), *p* = 0.25		
			
	PTB	cOR: 1.2 (0.5–2.9)	Exposed: 1 (1.6%), non-exposed: 0		
	PP				
	PA	cOR: 14.5 (1.5–140)			
	CS	cOR: 1.8 (1.1–3.2)			
Yi et al., 2020 [62]	PE	aOR: 1.10 (1.04–1.16)		44,428 exposed	Maternal age
	LBW	aOR: 1.64 (1.58–1.70)		1,893,996 non-exposed	
	PTB	aOR: 1.66 (1.59–1.73)			
	PP	aOR: 2.67 (2.54–2.82)			
	PA	aOR: 1.19 (1.05–1.35)			
	CS	aOR: 1.33 (1.30–1.35)			
	SB	aOR: 1.57 (1.44–1.70)			
	PPH	aOR: 1.10 (1.07–1.14)			

Abbreviations: aOR, adjusted odds ratio; APH, antepartum hemorrhage; aRR, adjusted risk ratio; ART, assisted reproductive technology; BMI, body mass index; CI, confidence intervals; cOR, crude odds ratio; cRR, crude relative risk; CS, cesarean section; DE, deep endometriosis; FSH, follicle-stimulating hormone; GA, gestational age; GDM, gestational diabetes mellitus; GH, gestational hypertension; ICSI, intra-cytoplasmatic sperm injection; IQR, interquartile range; IUGR, intrauterine growth restriction; IVF, in vitro fertilization; LBW, low birth weight; NS, non-significant; OE, ovarian endometriosis; PA, placental abruption; PAPP-A, Pregnancy Associated Plasma Protein A; PE, pre-eclampsia; PP, placenta previa; PPH, postpartum hemorrhage; PROM, premature rupture of membranes; PTB, preterm birth; SB, stillbirth; SES, socio-economic status; SGA, small for gestational age; SHiP, spontaneous hemoperitoneum. *Calculated cOR; ** 99.5% CI.

## Supplementary Materials

The following are available online at https://www.mdpi.com/2077-0383/10/4/667/s1, Supplementary Material S1: PRISMA checklist, Supplementary Material S2: Search strings, Supplementary Material S3: Explanation of Newcastle–Ottawa quality assessment scale, Figure S1: Forest plot for endometriosis and hypertensive disorders in pregnancy overall including all studies regardless of study quality, Figure S2: Funnel plot for endometriosis and hypertensive disorders in pregnancy overall, Figure S3: Forest plot for endometriosis and gestational hypertension including all studies regardless of study quality (a) and including only studies with spontaneous pregnancies (b), Figure S4: Funnel plot for endometriosis and gestational hypertension, Figure S5: Forest plot for endometriosis and pre-eclampsia including all studies regardless of study quality (a) and including only studies with spontaneous pregnancies (b), Figure S6: Funnel plot for endometriosis and pre-eclampsia, Figure S7: Forest plot for endometriosis and low birth weight including all studies regardless of study (a) and including only studies with spontaneous pregnancies (b), Figure S8: Funnel plot for endometriosis and low birth weight, Figure S9: Forest plot for endometriosis and small for gestational age including all studies regardless of study quality (a) and including only studies with spontaneous pregnancies (b), Figure S10: Funnel plot for endometriosis and small for gestational age, Figure S11: Forest plot for endometriosis and preterm birth including all studies regardless of study quality (a) and including only studies with spontaneous pregnancies (b), Figure S12: Funnel plot for endometriosis and preterm birth, Figure S13: Forest plot for endometriosis and placenta previa including all studies regardless of study quality (a) and including only studies with spontaneous pregnancies (b), Figure S14: Funnel plot for endometriosis and placenta previa, Figure S15: Forest plot for endometriosis and placental abruption including all studies regardless of study quality (a) and including only studies with spontaneous pregnancies (b), Figure S16: Funnel plot for endometriosis and placental abruption, Figure S17: Forest plot for endometriosis and cesarean section including all studies regardless of study quality (a) and including only studies with spontaneous pregnancies (b), Figure S18: Funnel plot for endometriosis and cesarean section, Figure S19: Forest plot endometriosis and stillbirth including for all studies regardless of study quality, Figure S20: Forest plot for endometriosis and postpartum hemorrhage including all studies regardless of study quality, Figure S21: Funnel plot for endometriosis and postpartum hemorrhage.
