# Supplementary material for: Endometriosis and Risk of Adverse Pregnancy Outcome: A Systematic Review and Meta-Analysis
Source: J Clin Med. 2021 Feb 9;10(4):667. doi: 10.3390/jcm10040667 (PMC7916165; doi:10.3390/jcm10040667)
Supplement: Supplementary file 1 [file jcm-10-00667-s001.zip › Supplementary Figures S1-S21.docx]

**Figure S1**: Forest plot for endometriosis and hypertensive disorders in pregnancy overall including all studies regardless of study quality.


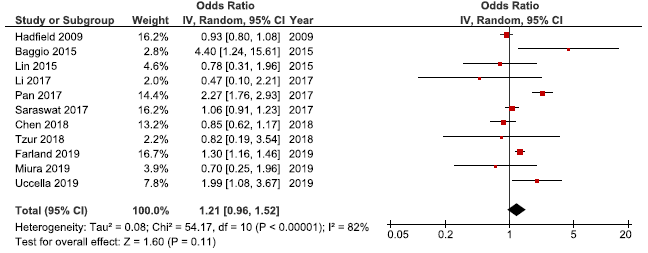


**Figure S2**: Funnel plot for endometriosis and hypertensive disorders in pregnancy overall.


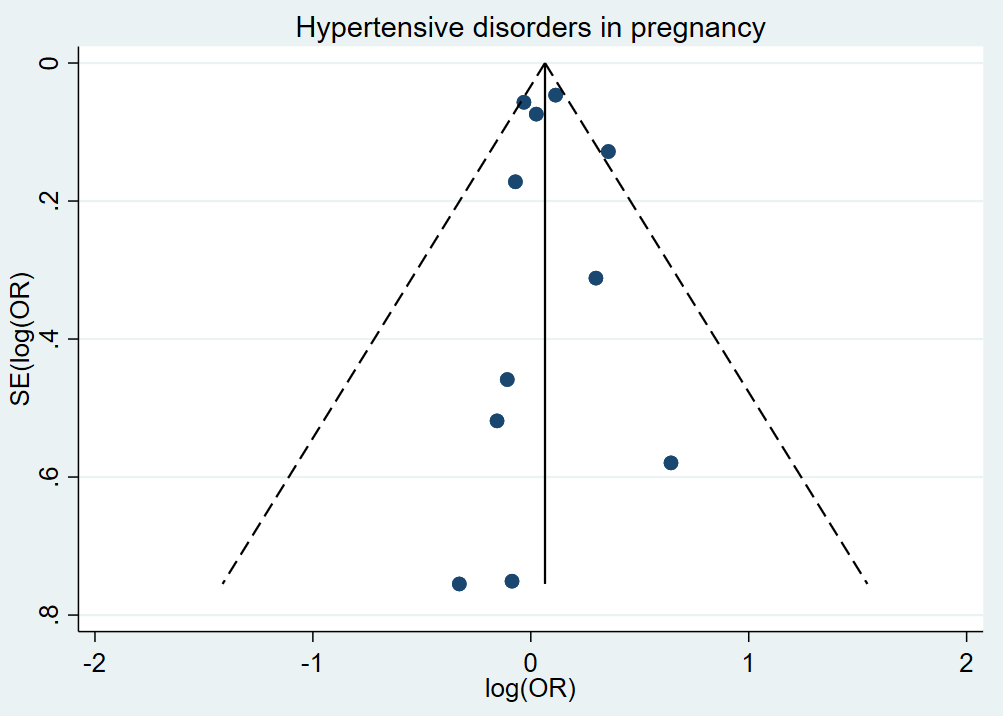


**Figure S3:** Forest plot for endometriosis and gestational hypertension including all studies regardless of study quality (a) and including only studies with spontaneous pregnancies (b).

(a)
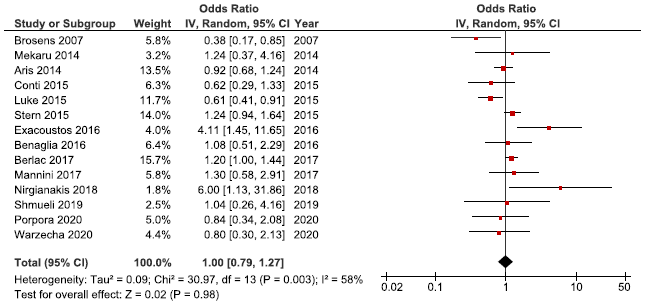


(b)


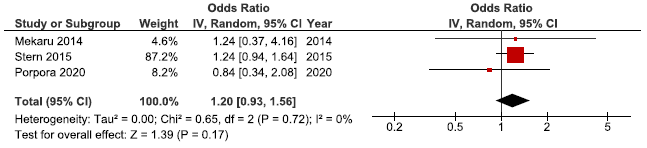


**Figure S4**: Funnel plot for endometriosis and gestational hypertension.


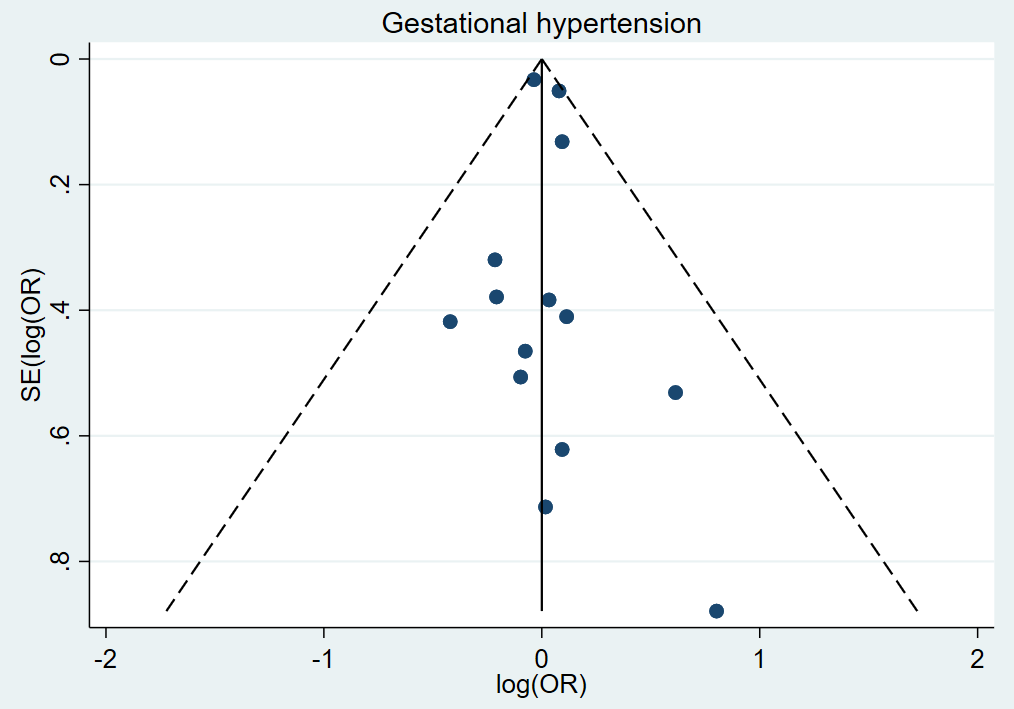


**Figure S5:** Forest plot for endometriosis and pre-eclampsia including all studies regardless of study quality (a) and including only studies with spontaneous pregnancies (b).

(a)
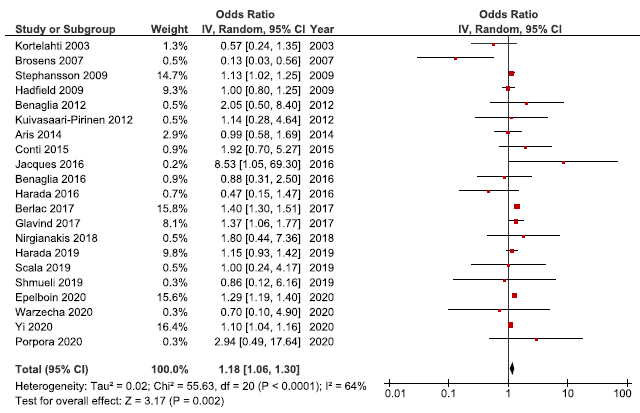


(b)


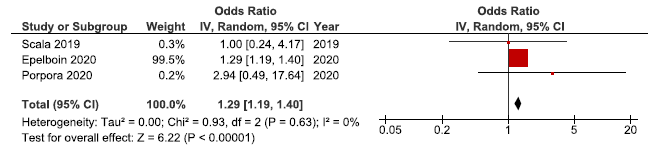


**Figure S6**: Funnel plot for endometriosis and pre-eclampsia.

**Figure S7:** Forest plot for endometriosis and low birth weight including all studies regardless of study (a) and including only studies with spontaneous pregnancies (b).

(a)


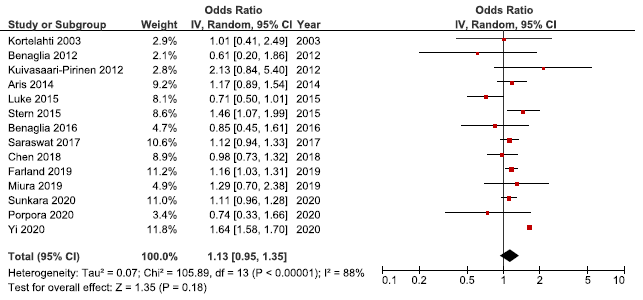


(b)


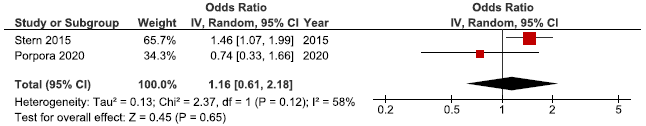


**Figure S8**: Funnel plot for endometriosis and low birth weight.

**Figure S9:** Forest plot for endometriosis and small for gestational age including all studies regardless of study quality (a) and including only studies with spontaneous pregnancies (b).

(a)
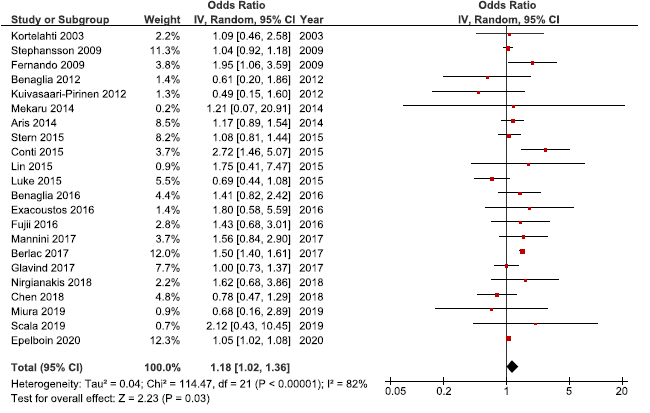


(b)


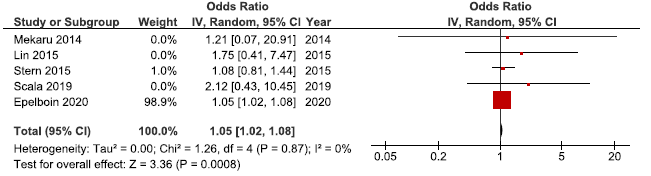


**Figure S10**: Funnel plot for endometriosis and small for gestational age.

**Figure S11:** Forest plot for endometriosis and preterm birth including all studies regardless of study quality (a) and including only studies with spontaneous pregnancies (b).

(a)
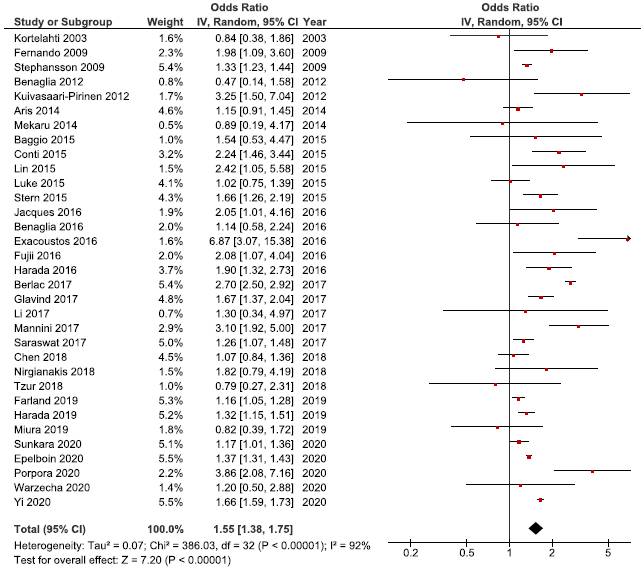


(b)


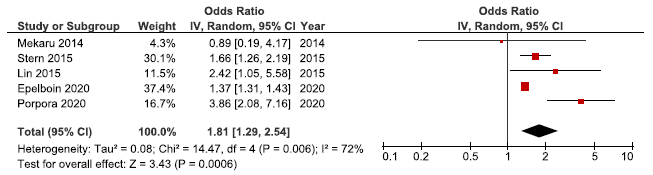


**Figure S12**: Funnel plot for endometriosis and preterm birth.

**Figure S13:** Forest plot for endometriosis and placenta previa including all studies regardless of study quality (a) and including only studies with spontaneous pregnancies (b).

(a)


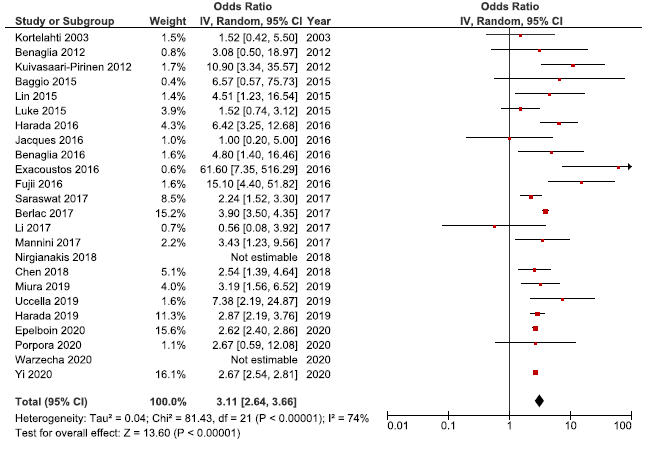


(b)


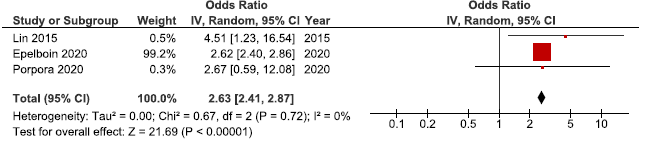


**Figure S14**: Funnel plot for endometriosis and placenta previa.

**Figure S15:** Forest plot for endometriosis and placental abruption including all studies regardless of study quality (a) and including only studies with spontaneous pregnancies (b).

(a)
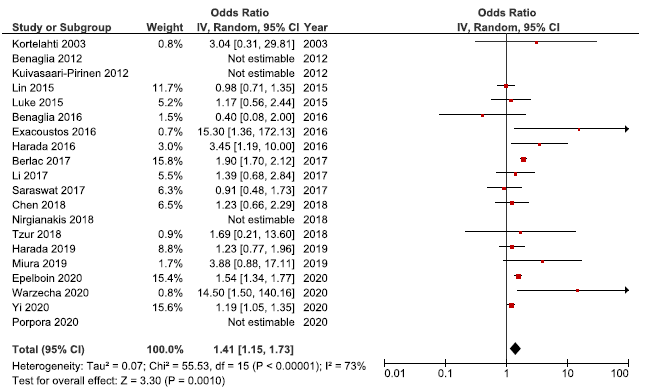


(b)


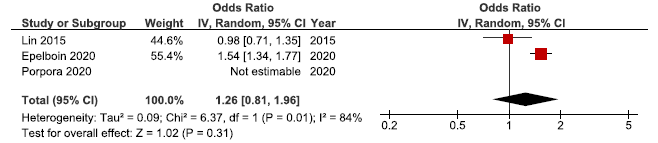


**Figure S16**: Funnel plot for endometriosis and placental abruption.

**Figure S17:** Forest plot for endometriosis and cesarean section including all studies regardless of study quality (a) and including only studies with spontaneous pregnancies (b).

(a)


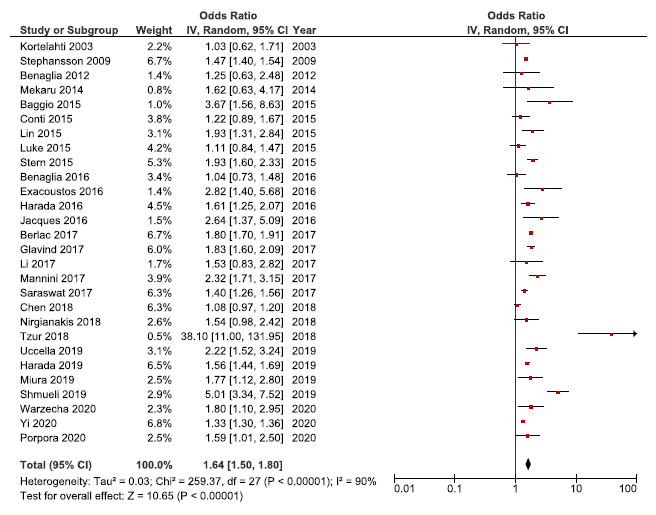


(b)


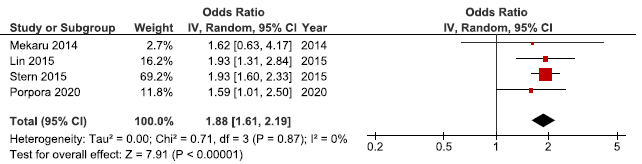


**Figure S18**: Funnel plot for endometriosis and cesarean section.

**Figure S19:** Forest plot endometriosis and stillbirth including for all studies regardless of study quality.


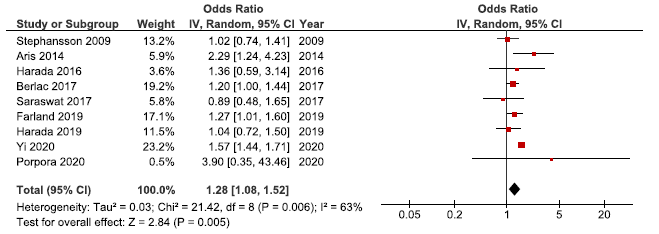


**Figure S20:** Forest plot for endometriosis and postpartum hemorrhage including all studies regardless of study quality.


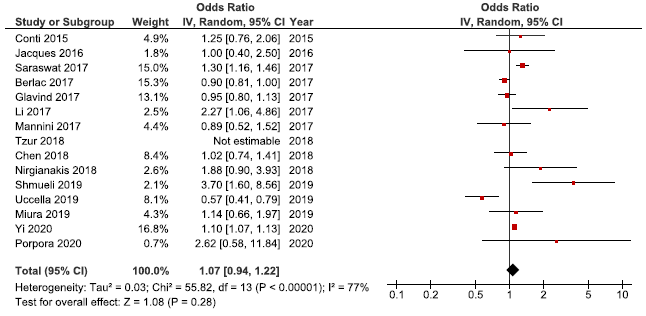


**Figure S21**: Funnel plot for endometriosis and postpartum hemorrhage.
