# Supplementary material for: Endometriosis and Risk of Adverse Pregnancy Outcome: A Systematic Review and Meta-Analysis
Source: J Clin Med. 2021 Feb 9;10(4):667. doi: 10.3390/jcm10040667 (PMC7916165; doi:10.3390/jcm10040667)
Supplement: Supplementary file 1 [file jcm-10-00667-s001.zip › Supplementary Material S2_search strings.docx]

Search string for PubMed

((((((((((((((((((((((((((("maternal hypertension") OR ("pregnancy induced hypertension")) OR ("postpartum hemorrhage")) OR (stillbirth)) OR ("c-section")) OR ("cesarean section")) OR ("intestinal perforation")) OR ("bowel perforation")) OR ("spontaneous hemoperitoneum")) OR ("placental abruption")) OR ("placenta previa")) OR ("antepartum hemorrhage")) OR ("gestational age")) OR ("premature labor")) OR ("premature delivery")) OR ("premature birth")) OR ("preterm labor")) OR ("preterm delivery")) OR ("preterm birth")) OR ("low birth weight")) OR ("Infant, Very Low Birth Weight"[Mesh]) OR ("Infant, Extremely Low Birth Weight"[Mesh]) OR ("small for gestational age")) OR ("hypertensive disorders in pregnancy")) OR ("gestational hypertension")) OR (preeclampsia)) OR (pre-eclampsia)) OR ((((((((((((("Postpartum Hemorrhage"[Mesh]) OR ("Stillbirth"[Mesh])) OR ("Cesarean Section"[Mesh])) OR ("Intestinal Perforation"[Mesh])) OR ("Hemoperitoneum"[Mesh])) OR ("Abruptio Placentae"[Mesh])) OR ("Placenta Previa"[Mesh])) OR ("Gestational Age"[Mesh])) OR ("Premature Birth"[Mesh])) OR ("Infant, Low Birth Weight"[Mesh])) OR ("Infant, Small for Gestational Age"[Mesh])) OR ("Hypertension, Pregnancy-Induced"[Mesh])) OR ("Pre-Eclampsia"[Mesh]))) AND ((endometriosis) OR ("Endometriosis"[Mesh]) OR (adenomyosis) OR ("Adenomyosis"[Mesh])) NOT (casereports[Filter] OR comment[Filter] OR editorial[Filter] OR letter[Filter]))

Search string for EMBASE

('preeclampsia'/exp OR 'maternal hypertension'/exp OR 'small for date infant'/exp OR 'low birth weight'/exp OR 'premature labor'/exp OR 'gestational age'/exp OR 'antepartum hemorrhage'/exp OR 'placenta previa'/exp OR 'hemoperitoneum'/exp OR 'intestine perforation'/exp OR 'cesarean section'/exp OR 'stillbirth'/exp OR 'postpartum hemorrhage'/exp OR 'pregnancy induced hypertension' OR 'postpartum hemorrhage' OR stillbirth OR 'c-section' OR 'cesarean section' OR 'intestinal perforation' OR 'bowel perforation' OR 'spontaneous hemoperitoneum' OR 'placental abruption' OR 'placenta previa' OR 'antepartum hemorrhage' OR 'gestational age' OR 'premature labor' OR 'premature delivery' OR 'premature birth' OR 'preterm labor' OR 'preterm delivery' OR 'preterm birth' OR 'low birth weight' OR 'small for gestational age' OR 'hypertensive disorders in pregnancy' OR 'gestational hypertension' OR preeclampsia OR 'pre eclampsia') AND ('endometriosis' OR 'endometriosis'/exp OR adenomyosis OR 'adenomyosis'/exp) NOT 'case report'/de NOT ('editorial'/it OR 'letter'/it OR 'note'/it)
