# Supplementary material for: Endometriosis and Risk of Adverse Pregnancy Outcome: A Systematic Review and Meta-Analysis
Source: J Clin Med. 2021 Feb 9;10(4):667. doi: 10.3390/jcm10040667 (PMC7916165; doi:10.3390/jcm10040667)
Supplement: Supplementary file 1 [file jcm-10-00667-s001.zip › Supplementary Material S3_Explanation of Newcastle-Ottawa quality assessment scale.docx]

**Explanation of Newcastle-Ottawa quality assessment scale**

**For case-control studies**

**Selection**

1. *Is the case definition adequate*

A * can be granted if the case consisting of one of the outcomes investigated (gestational hypertension, pre-eclampsia, low birth weight, small for gestational age, gestational age, preterm delivery, antepartum hemorrhage (including placenta previa or placental abruption), spontaneous bowel perforation in pregnancy, spontaneous hemoperitoneum in pregnancy, cesarean section, stillbirth or postpartum hemorrhage) is identified through medical records or with self-reports supported by verification in medical records.

1. *Representativeness of the cases*

To be granted a * if the percentage of included cases with at least one of the investigated outcomes is high. The participation rate should be at least 80%.

1. *Selection of controls*

Children born without the outcome of interest represents controls. Since most pregnancies involve contact to the hospital at some point and most births are at the hospital, in most/all cases the studies can receive a *.

1. *Definitions of controls*

Since controls are composed of women with children born without any of the outcomes. It is possible for the woman to have had a previous child born with one or more of the outcomes but that does not have reference to the current pregnancy. As a result, the controls can be granted a *.

**Comparability**

1. *Comparability of cases and controls on the basis of the design or analysis*

A * can be granted if there is adjusted, stratified or matched by at least two of the following: maternal age, smoking, BMI and socio-economic status.

If all the mentioned are controlled for two ** can be given.

Adjustment or stratification for ART will not result in a *.

**Exposure**

1. *Ascertainment of exposure*

Assessment of exposure can give a * if endometriosis is diagnosed by either histology, surgery, imaging or International Classification of Diseases (ICD)-8(625.3*) and -10(N80*). Diagnosis based on symptoms alone will not result in a *.

1. Studies will receive a * if there is no difference in how information was gathered in the case and in the control group.
2. *Non-response rate*

To get a * the non-responsive rate in the study must be reasonably equal between cases and controls. A * will be granted if the difference in response-rate between the two groups is less than 20%.

For cohort studies

**Selection**

1. *Representativeness of the exposed cohort*

Women with endometriosis must represent the average woman of fertile age for the study to granted a *.

This means women in fertility treatment are not representative of the average population.

1. *Selection of the non-exposed group*

Women without endometriosis must derive from the same population as the exposed group to be given a *.

1. *Ascertainment of exposure*

Assessment of exposure can give a * if endometriosis is diagnosed by either histology, surgery, imaging or International Classification of Diseases ICD)-8(625.3*) and -10(N80*). Diagnosis based on symptoms alone will not result in a *.

1. *Demonstration that outcome of interest was not present at start of study*

Due to all outcomes occurring during pregnancy they will not be present in the beginning of the study thus all studies can be granted a *.

**Comparability**

1. *Comparability of cohorts on the basis of the design or analysis*

A * can be granted if there is adjusted, stratified or matched by at least two of the following: maternal age, smoking, BMI and socio-economic status.

If all the mentioned are controlled for two ** can be given.

Adjustment or stratification for ART will not result in a *.

**Outcome**

1. *Assessment of outcome*

A * can be granted if assessment of the outcomes is obtained from medical records or with self-reports supported by verification in medical records.

1. *Was follow-up long enough for the outcomes to occur*

Studies will be gives a * if follow-up is long enough for the outcomes to occur. All outcomes will occur during pregnancy or at the time of birth thus follow-up until birth is adequate and will result in a *.

1. *Adequacy of follow-up of cohorts*

Studies will receive a * if at least 80% of participants complete the follow-up period.
